# Tactile Sensing for Minimally Invasive Surgery: Conventional Methods and Potential Emerging Tactile Technologies

**DOI:** 10.3389/frobt.2021.705662

**Published:** 2022-01-07

**Authors:** Wael Othman, Zhi-Han A. Lai, Carlos Abril, Juan S. Barajas-Gamboa, Ricard Corcelles, Matthew Kroh, Mohammad A. Qasaimeh

**Affiliations:** ^1^ Engineering Division, New York University Abu Dhabi, Abu Dhabi, United Arab Emirates; ^2^ Mechanical and Aerospace Engineering, New York University, New York, NY, United States; ^3^ Digestive Disease Institute, Cleveland Clinic Abu Dhabi, Abu Dhabi, United Arab Emirates; ^4^ Digestive Disease and Surgery Institute, Cleveland Clinic Main Campus, Cleveland, OH, United States; ^5^ Cleveland Clinic Lerner College of Medicine, Cleveland, OH, United States

**Keywords:** MEMS, sensors, surgery, laparoscopic, minimally invasive surgeries, robotic, tactile

## Abstract

As opposed to open surgery procedures, minimally invasive surgery (MIS) utilizes small skin incisions to insert a camera and surgical instruments. MIS has numerous advantages such as reduced postoperative pain, shorter hospital stay, faster recovery time, and reduced learning curve for surgical trainees. MIS comprises surgical approaches, including laparoscopic surgery, endoscopic surgery, and robotic-assisted surgery. Despite the advantages that MIS provides to patients and surgeons, it remains limited by the lost sense of touch due to the indirect contact with tissues under operation, especially in robotic-assisted surgery. Surgeons, without haptic feedback, could unintentionally apply excessive forces that may cause tissue damage. Therefore, incorporating tactile sensation into MIS tools has become an interesting research topic. Designing, fabricating, and integrating force sensors onto different locations on the surgical tools are currently under development by several companies and research groups. In this context, electrical force sensing modality, including piezoelectric, resistive, and capacitive sensors, is the most conventionally considered approach to measure the grasping force, manipulation force, torque, and tissue compliance. For instance, piezoelectric sensors exhibit high sensitivity and accuracy, but the drawbacks of thermal sensitivity and the inability to detect static loads constrain their adoption in MIS tools. Optical-based tactile sensing is another conventional approach that facilitates electrically passive force sensing compatible with magnetic resonance imaging. Estimations of applied loadings are calculated from the induced changes in the intensity, wavelength, or phase of light transmitted through optical fibers. Nonetheless, new emerging technologies are also evoking a high potential of contributions to the field of smart surgical tools. The recent development of flexible, highly sensitive tactile microfluidic-based sensors has become an emerging field in tactile sensing, which contributed to wearable electronics and smart-skin applications. Another emerging technology is imaging-based tactile sensing that achieved superior multi-axial force measurements by implementing image sensors with high pixel densities and frame rates to track visual changes on a sensing surface. This article aims to review the literature on MIS tactile sensing technologies in terms of working principles, design requirements, and specifications. Moreover, this work highlights and discusses the promising potential of a few emerging technologies towards establishing low-cost, high-performance MIS force sensing.

## 1 Introduction

Minimally invasive surgery (MIS) has changed surgical practices during the last three decades, and it has attracted the attention of many researchers who are trying to contribute to its development. MIS procedures are achieved through small incisions (0.3–1 cm) or natural orifices such as the mouth, nose, urethra, vagina, and anus. MIS instruments are characterized by small size, flexibility, precision, and reliability (Hindle and Hindle, 2001; Kelley, 2008).

MIS includes numerous advantages such as reduced postoperative pain, shorter hospital stay, decreased surgical site infections, and faster recovery time. MIS approaches have now become the gold standard of several common procedures in our daily practice, including appendectomy, cholecystectomy, and hernia repairs (Litynski, 1999). Such revolutionary advances would not have been made possible without the development of improved instruments, anesthesia, and advanced optical methods (Lane, 2018). In an attempt to improve current techniques and technologies, the concept of minimally invasive robotic surgery (MIRS) was introduced, where surgeons operate medical robots to perform MIS procedures. The MIRS offers increased dexterity to surgeons who wish to perform complex cases, even in reduced anatomical spaces, with greater precision and accuracy. Undesirable, yet inevitable, vibrations and tremors that would usually come from the surgeon’s hands in MIS are also eliminated altogether (Fuchs, 2002). Today, it remains the most popular method used to detect cancer through a procedure called palpation, in which surgeons indirectly feel tissues in an attempt to determine the presence of harder, stiffer tumor cells (Krouskop et al., 1998).

**FIGURE 1:**
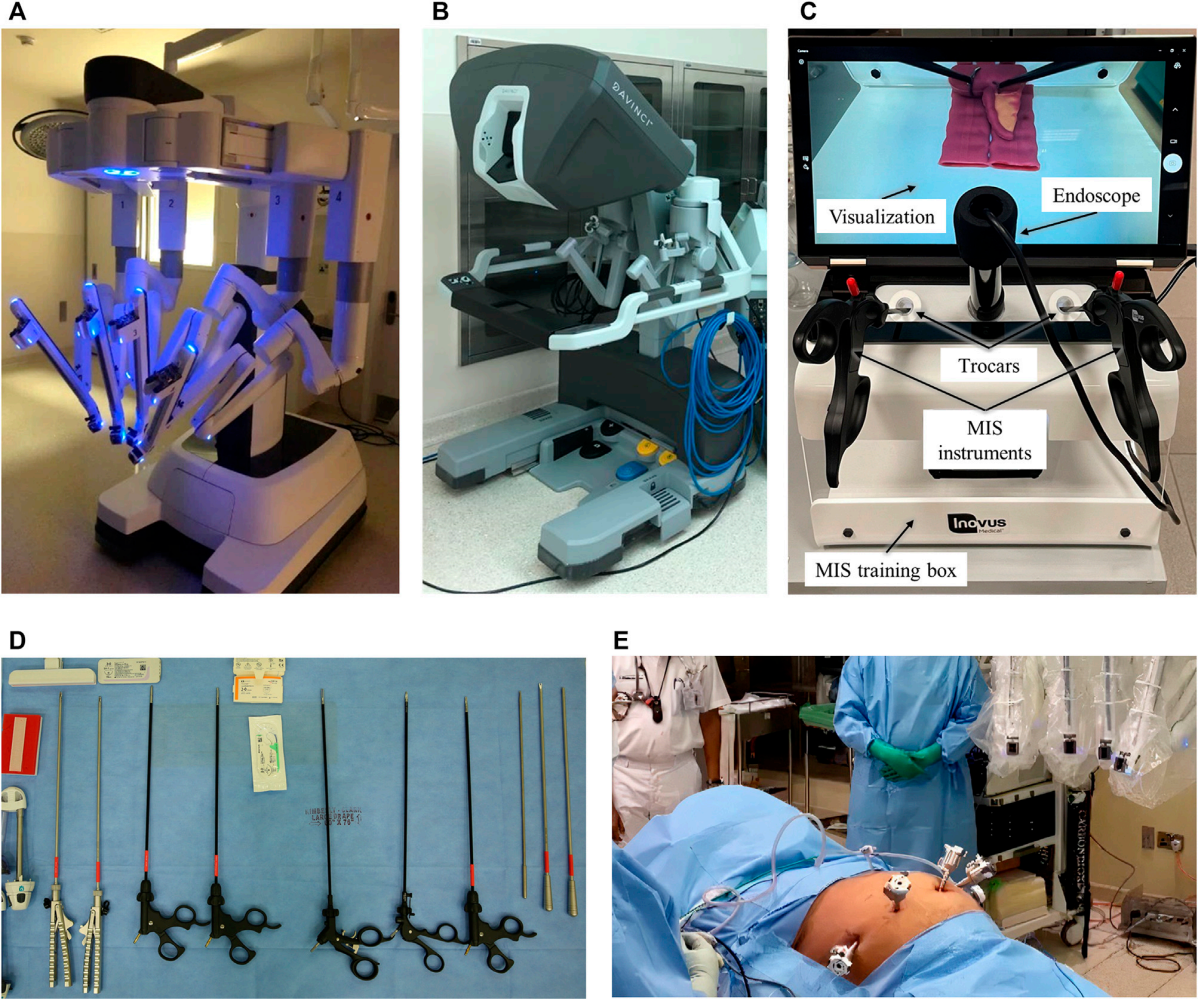
Minimally Invasive Surgery Systems. **(A,B)** Da Vinci surgical robotic system for MIS: **(A)** patient cart holding the camera and instruments that the surgeon controls remotely and **(B)** surgeon console that controls the instrumented arms and provides a high-definition 3D view of the operation site. **(C)** Laparoscopic box trainer with an endoscope and surgical instruments used for MIS simulations. **(D)** Surgical instruments used in laparoscopic surgery and training. **(E)** Laparoscopic operation with port accesses in the abdominal cavity and the docking of the robot arm with the ports.

The recent advancements in robotics and control systems are pushing MIS one step further. As an example, the da Vinci robotic surgical system, shown in Figures 1A,B, was launched in the late 90s and is currently being used around the world in various surgeries, offering 3D immersive vision, motion scaling, and simplification of otherwise complex movements (Ballantyne and Moll, 2003; Palep, 2009). Further developments in MIRS and telecommunications are also making telesurgery possible, in which surgeons can perform computer-driven surgeries remotely from different locations (Marescaux et al., 2001; Alderson, 2019). On the other hand, the implementation of the MIRS has encountered several limitations mainly associated with the high costs of operations. The initial investment is very high, usually ranging from $1 million to $2.5 million, shooting up the overall operational cost per case to $3,200 on average (Morgan et al., 2005; Turchetti et al., 2012). Eventually, an average of 150–250 procedures must be performed in order for a surgeon to become proficient in operating the robot (Barbash and Glied, 2010).

From the medical perspective, the lack of sense of touch when using MIRS is a major challenge. Historically, open surgery allowed surgeons to have direct contact with the tissue and preserve the touch sense. With the introduction of laparoscopic surgery (LS), this sense was partially affected but completely eliminated with MIRS. The commonly used robotic surgical systems, such as the da Vinci system, has no touch feedback; nonetheless, the sense of touch is essential for safely maneuvering organs, tissues, and sutures. Haptic, or touch-based, interactions offer more reliable determination of the consistency of the tissues, preventing surgeons from accidentally applying excessive forces and damaging them (Sastry et al., 1997). Additionally, grasping force feedback helps to prevent tissue slippage, hence enhancing MIS time efficiency. Therefore, a critical necessity is to develop sensorized MIS tools that bio-mimic the human finger’s ability to detect normal and shear forces, tissues’ softness, and other physical properties. Furthermore, MIS simulations, such as the one shown in Figure 1C, become more effective for trainees when combined with force feedback systems. Training with sensorized instruments provides awareness of the forces being applied and the tissues being grasped, leading to a shorter learning curve with a steadier upward trend (Overtoom et al., 2019).

The two essential components to bring the sense of touch back into MIS include tactile force sensing of the instruments and haptic feedback to the surgeon. In this review, we will solely focus on discussing the different types of sensors used in tactile force sensing and their respective recent developments within MIS. In the literature, several interesting reviews targeted the general biomedical applications while touching on the MIS and RMIS fields (Tiwana et al., 2012; Al-Handarish et al., 2020). Some other reviews focused more on MIS conventional tactile sensing technologies (Eltaib and Hewit, 2003; Konstantinova et al., 2014; Bandari et al., 2020). Further, a recent review paper on tactile perception in MIS is focused on the algorithms utilized by the tactile sensing systems to evaluate the data rather than discussing the sensing principles of sensors (Huang et al., 2020). To this end, this work is an up-to-date comprehensive review, attacking from a historical progress point of view, discussing conventional methods in a balanced manner, comparing the pros and cons of all methods, and highlighting emerging technologies that could potentially contribute heavily to the MIS tactile sensing field.

The history of MIS and the requirements for implementing tactile sensors with surgical tools are highlighted in Sections 2 and 3, respectively. Following this, MIS-oriented studies concerning two conventional tactile sensing approaches, i.e., electrical and optical sensing, are discussed in Section 4. In Section 5, the potential of emerging tactile sensors, i.e., microfluidic and imaging sensors, as promising candidates for developing sensorized MIS tools is presented. Towards the end, current technological obstacles and perspective outlooks are summarized in the concluding remarks.

## 2 Minimally Invasive Surgery

“Surgeons applaud large incisions and denigrate “keyhole surgery.” Patients, in contrast, want the smallest wound possible, and we at Britain’s first department of minimally invasive surgery are convinced that patients are right,” John Wickham, who first coined the term minimally invasive surgery (MIS), wrote in an article in British Journal of Surgery published in 1987 (Hindle and Hindle, 2001).

Over the past five decades, the evolution of the surgical field has exceeded the expectations in terms of clinical outcomes, from large incision/open procedures in the 1950s to MIS in the late 1990s, followed by the revolution of the MIRS in the late 2000s. As a result of the availability of advanced technologies and new surgical tools in the market, the development of novel surgical approaches has been the target of different surgical groups worldwide. As mentioned above, MIS’s concept encompasses all the diagnostic and/or therapeutic techniques accessing different anatomical cavities, organs, and tissues through natural orifices or small incisions (Kelley, 2008), for example, the access of the abdominal cavity in order to remove the gallbladder (laparoscopy), the access of the chest for a lung nodule removal (thoracoscopy), the access to the knee for a ligament repair (arthroscopy), and the access to the colon in order to remove a polyp (colonoscopy). The commonly used surgical instruments in laparoscopic surgery and training are shown in Figure 1D.

The approach has many advantages, such as less postoperative pain, fewer surgical incisions, shorter hospital stay, better postoperative recovery time, and lower risk of surgical site infections (Hindle and Hindle, 2001; Kelley, 2008). Its use has not been limited to the field of general surgery, whereas urologists, gynecologists, cardiovascular surgeons, thoracic surgeons, vascular surgeons, and other specialties have also taken advantage of the evolution of these surgical systems.

The earliest record of endoscopy, or the practice of introducing instruments into the body to view internal organs, was introduced by Hippocrates (460–370BC), the “Father of Medicine.” Modern endoscopy only began with the advent of light conductors used to illuminate body parts (1853), as well as tubes used to extract fluids and ascites from the body (1938) (Radojcić et al., 2009). However, the profound development of these minimally invasive techniques started in the early 1970s when Shinya and Wolfe reported the first experiences of removing colon polyps using rigid colonoscopes, procedures characterized by minimal morbidity and mortality. These were the formal beginnings of a new era called “endoscopic surgery.” With time, this practice became solidly and routinely established by numerous gastrointestinal surgeons. Posteriorly, the LS was consolidated in the late 1980s with the incorporation of video laparoscopy, a technology developed since the 1960s by various groups of gynecologists and urologists in Germany. Its most prominent and remembered leader was Kurt Semm. The first case of LS successfully reported in the medical literature was in 1987 by the French surgeon Phillip Mouret. The procedure performed was a laparoscopic cholecystectomy. Two years later, in 1989, the American College of Surgeons (ACS) endorsed this new surgical procedure with the support of other leaders in the surgical field, Eddie Reddick and Douglas Olsen (Kelley, 2008).

Almost two decades ago, using the same principles, the concept of endoscopic surgery through natural orifices was established by its acronym “NOTES.” A considerable number of surgeries were developed using a combination of endoscopic and laparoscopic instruments, with the aim of accessing the abdominal cavity and removing organs without external scars. Its most important leaders were Kalloo and Kantsevoy. These concepts became relevant when they were scientifically accepted by the American Society of Gastrointestinal and Endoscopic Surgeons (SAGES) and the American Society of Gastrointestinal Endoscopists (ASGE). Then, several other procedures were developed: transoral appendectomy, transvaginal cholecystectomy, transvaginal nephrectomy, and transvaginal gastric sleeve. Although the difficulties in standardizing the techniques limited their popularity at the time, they still played a significant role in the evolution of MIS (Litynski, 1999).

More recently, MIRS emerged with the aim of offering various advantages over traditional LS, such as three-dimensional vision, greater dexterity, improved mobility, usage of articulated instruments, increased range of movements, reduced tremor, and better ergonomic position for the surgeon (Lane, 2018). Figure 1E shows a robot-assisted laparoscopic operation with port accesses in the abdominal cavity and the robot arms docking with the ports. MIRS history began with the Puma 560 robot, used by Kwoh to perform neurosurgical biopsies with greater precision in 1988 (Lanfranco et al., 2004). For gastrointestinal surgery, the big step was taken in 2001 when Marescaux performed the first transcontinental robotic cholecystectomy, where the surgeon was based in New York (United States) using the ZEUS surgical system and the patient was on the operating room table in Strasbourg (France) (Marescaux et al., 2002). Subsequently, the da Vinci surgical system was positioned as the most complete and developed robotic platform with the endorsement of the Federal Agency for the Administration of Food and Drug Administration (FDA) in 2000. The future of the MIS will be influenced by several factors, including the development of new surgical instruments with better performance. The field of research is advancing by leaps and bounds in order to provide the patient with the best possible clinical outcomes (Alderson, 2019).

## 3 Tactile Sensing in Minimally Invasive Surgery

In the modern era, the increased interaction between humans and technological devices has motivated the development of several sensing devices, e.g., temperature (Moser and Gijs, 2007), humidity (Han et al., 2012), accelerometers (James et al., 2004), and gas sensors (Tit et al., 2018; Shaheen et al., 2020). Recently, tactile sensing has gained significant interest due to its potential impact on MIS grasping and manipulation, among other applications. Ever since MIS has become mainstream within the medical community, many proofs of concept and sensor-integrated instruments have been attempted and tested. Despite the hundreds of studies and research efforts to integrate tactile sensation and haptic information in MIS, to this day, no commercialized product has been established in the mainstream. However, this is not to say that significant progress has not been made to put the sense of touch back into the hands of surgeons. By looking into the history of MIS, it is evident that tactile sensing has been a challenging task, and recently its developments have dramatically escalated.

As stated earlier, the first LS through a minimal incision dates back to the 1970s. This was about the time when tactile sensing for applications in robotics first emerged, intending to allow machines to receive and respond to force input (Tegin and Wikander, 2005). In the same year that video laparoscopy was introduced (1982), one of the first robotic-application tactile sensing reviews was published by Harmon, highlighting present and future outlooks of tactile sensing and its potential in the field. Harmon singled out three tactile sensing fields that require major development across all criteria: prosthetics, medical examination, and surgery, all of which are medical applications (Harmon listed industry, space, underwater, assembly, and other applications as less difficult and better developed) (Harmon, 1982). He also noted that those three fields are in high demand for decent spatial and time resolution, force sensitivity, range, and complex pattern recognition, giving the applications a 5 out of 5 on the “demanding scale.” It is made clear that starting from the 1980s, tactile sensing for medical applications was a field in need of development.

Advanced robotic grippers with integrated force and torque sensors, laser range detectors, actuators, and communication electronics emerged during the 1980s (Dietrich et al., 1990). Although Harmon’s desire for automation in MIS was never realized, several developments were made in manufacturing tactile sensor arrays, miniaturization, and new designs specifically targeted towards detecting tissue properties during the 1990s and 2000s. Several studies were performed to prove the advancements and potential for robotic grippers in MIS. For instance, Trejos et al. (2008) showed that robot-conducted palpation led to a 35% decrease in maximum applied force and a 50% increase in detection accuracy of tumors, as well as an improved completion time.

In 1999, the World Health Organization established a new protocol regarding proper cleaning and sterilization of medical instruments with the possibility of being subjected to prion contamination (World Health Organization, 2000). This guideline mandates tools to be sterilized with sodium hydroxide or sodium hypochlorite—both corrosive chemicals pose a threat to the involved electronics and circuits (Trejos et al., 2008). The new requirement sets a new standard for all MIS sensors and will ensure that any MIS-ready tactile sensing device must withstand sterilization. It is worth noting that these issues that once hindered the development of tactile sensing in MIS remain obstacles. Miniaturization while preserving sensitivity and range and resistance to flexing and sterilization and being easily manufactured and disposable are still issues and trade-offs that affect modern designs. While many researchers have designed elaborate systems that help with tasks such as ranking stiffness, discriminating organs, and determining tissue properties, such as the feedback endoscopic surgical grasper developed by Rosen et al. (1999), few have demonstrated technology that is affordable and easily usable. Just as important as functionality, making a force and tissue sensor universally affordable and easily usable without specialized training is key to a successful design.

Over recent years, more attention has been given to processing force data and presenting the feedback to surgeons. While many have traditionally used visual displays to warn of tissue irregularities detected by laparoscopic devices, others have experimented with vibrational, auditory, or temperature cues. For example, Yao (2004) investigated using an arthroscopy hook with an accelerometer that amplified forces and vibrations through an actuator on the handle. When coupled with auditory feedback, tear detection was improved in an experiment. Other methods, such as tactile displays, have also been explored but with little success. In another demonstration by King et al. (2009a), the addition of a tactile feedback system has substantially decreased the grip force when performing the same task. This has implications in preventing damage made to grasped tissues and improving the overall control and maneuverability of devices. Documentation has proven that the combined effort in developing tactile sensing and feedback is important for providing haptic guides to surgeons.

Typical tactile sensors, regardless of their transduction technology, consist of three major components. The first is the sensing unit that converts pressure to a quantifiable signal. Common transducer technologies used in MIS include piezoresistive, piezoelectric, capacitive, optical, and elastomeric technologies (Bandari et al., 2020). Depending on the application, individual sensing elements can be arranged into one- or two-dimensional arrays, distributed across a continuous plane. For instance, palpation requires the measurement of relative hardness variations across a tissue, requiring a two-dimensional cluster of individual sensing elements (Naidu et al., 2016). Then comes an electronics component that contains specific circuits to process, filter, and interpret data (Eltaib and Hewit, 2003). The final component offers either rigid or flexible supports and protection, including waterproofing, heatproofing, and shock-proofing elements. However, it is crucial that this layer does not interfere with the operation of the sensing elements, produce excessive noise and inaccuracies, or cause a significant change in the stiffness of the structure.

The current structure of MIS tools has guided the development of MIS force sensing systems. Several researchers have evaluated the integration of force sensors at different locations on MIS graspers and probes. Few attempts proposed having the sensors outside the patient’s body to simplify the measurement of forces, as the size and sterilization requirements are not involved (Hanna et al., 2008). In some other attempts, installing sensors on the shaft of the tool allowed for measuring kinesthetic forces acting at the tip of the instrument (Berkelman et al., 2003). As an advantage of such indirect force measurements, contact forces are acquired without compromising the contact surface of the tool. However, the accuracy and precision of the indirect measurement remain questionable due to the influence of friction at the entry point and driving mechanism (Shimachi et al., 2004). Also, forces acting on the tissue are not exactly represented by forces acting on the handle of the instrument (Trejos et al., 2008). On the other hand, the direct measurement technique of contact forces at the tissue-tool level has been proven more precise and accurate (Özin et al., 2019). Sensors placed on the grasping tips are capable of accurately measuring kinesthetic and tactile forces in real-time. The contact area and pressure distribution, as well as the pressure center, can only be measured directly. Furthermore, the direct force measuring technique is not affected by the friction of the driving mechanism and is solely dependent on the interaction at the end-effector of the tool. Nevertheless, the addition of a tactile sensor, e.g., a thin-film layer, between the instrument and the tissue alters the tool characteristics by a certain amount. The debate on what location is the most suitable for MIS force sensors integration is still active, where a combination of different sensing locations might bring up more conclusive force measurements in a trade-off with the overall cost of the sensorized surgical instrument.

Different types of forces are involved when considering tactile sensing for MIS. Information about forces, whether being measured at the tip, the rod, or the base of the tool, can be used to restore the grasping, manipulation, and displacement actions performed by the surgeon. The most important and straightforward type of force to measure is the normal force that can provide an estimation of the applied pressure on the grasped or palpated tissue. Measuring static normal forces can prevent tissue damage from an overwhelmed grasping and manipulation (Tholey et al., 2004). On the other hand, meaningful information about tissue biomechanics, e.g., stiffness, can be obtained through dynamic loading. With the aid of MIS techniques, the detection of tumorous regions within the tissue is achieved through dynamic palpation (Krouskop et al., 1998). The shear force, also referred to as friction force, occurred to be important in preventing tissue slippage from the tool and maintaining the tissue in the safe zone without damage (Khadem et al., 2016). It was validated that, through estimating shear forces, sensorized MIS tools can provide both stability and robustness to the grasping action and improve the efficiency of the operation. Normal and shear force can also be measured on the different spots of the tool to detect any kind of undesired pressing or friction with nearby organs. In situations where blood vessels are present, they can be avoided by sensing the weak periodic pressures caused by pulses.

Before discussing each respective tactile sensing method, it is worth examining the sensor requirements associated with tactile sensing in MIS. The list of requirements, presented in Table 1, acts as criteria determining how appropriate and effective the sensor is within MIS procedures.

**TABLE 1:** List of sensor requirements for MIS tactile sensing.

Category	Requirement	Description
Operational requirements	Sensitivity	Produce accurate data with at least 0.2 N sensitivity for MIS (Lazeroms et al., 1996)
	Dynamic range	Typical medical forces range between ±10 N, laparoscopic surgical tools apply forces between 0 and 25 N but can go as high as 40 N (Kalantari et al., 2010)
	Frequency	Typical laparoscopic grasping frequencies do not exceed 3 Hz (Sarmah and Gulhane, 2010)
	Repeatability/linearity	Produce repeatable, precise data without drift and hysteresis error in differing environments
	Dexterity	Cannot sacrifice or interfere with surgeon dexterity (by being too bulky, fragile, rigid)
	Response rate	Provide rapid, on-the-fly measurements (within 1 millisecond) (Yousef et al., 2011)
Hardware requirements	Miniaturization	Needs to fit within laparoscopic width of 5–8 mm in a typical MIS tool
	Reliability	Robust, functional through entire surgical operation, reducing moving parts in sensor usually increases reliability
	Waterproofing	Needs to be resistant in bodily environments of bodily fluids, organs, and soft tissues
	Compatible with MIS tools	Cannot interfere and be interfered with the operation of endoscopes, catheters etc.
	Sterilizable	Needs to be easily sterilizable for MIS to avoid contamination of infection (needs to be stable in acidic and basic sterilizing environments)
External requirements	Cost	Laparoscopic tools are thrown out after each operation, so they need to be disposable and affordable
	Assembly	Needs to be easily assembled and integrated within a wide range of MIS models and tools, will also become beneficial for mass production purposes
Recommended requirements	Working area	Wide working area to allow force measurement across a laparoscopic grasper or tool
	Compliance measurements	Measurement of hardness and softness of tissues through the force sensor
	Force identification	Differentiation between normal and shear forces helps with tissue characterization, surface friction, viscosity (Yousef et al., 2011)
	Temporal variation	Differentiate between dynamic and static forces, measure both accurately (Yousef et al., 2011)

## 4 Conventional Tactile Sensing Technologies

Tactile sensors for MIS and MIRS applications should be capable of estimating the magnitude, direction, and location of the applied force on the contact surface. Additionally, evaluating the compliance and texture of the grasped organs and detecting slippage are fundamental requirements for increasing the efficiency of the medical practice. In order to facilitate these capabilities, several studies attempted to integrate tactile sensors that bio-mimic the human tactile system with MIS surgical instruments. This section discusses the conventional tactile sensors designated for MIS, which are mainly silicon-based devices fabricated using the micro-electro-mechanical systems (MEMS) technology, and developed based on electrical or optical tactile sensing principles.

### 4.1 Piezoresistive Tactile Sensing

Piezoresistive force sensors rely on the piezoresistive effect, wherein applied mechanical forces lead to measurable changes in the electrical resistance of the sensing element (Tiwana et al., 2012). This type of force sensor utilizes a piezoresistive component, usually a metal, conductive elastomer, or semiconductor such as a silicone substrate, which deforms or distorts in structure under the application of pressure (Yousef et al., 2011). Such deformations can either increase or decrease the electrical resistance of the sensing material according to its geometry and orientation. The value of the resistance, R , is given by the following:

$$R=\frac{\mathrm{\rho L}}{S},$$

where ρ is the resistivity, L is the length, and S is the cross-sectional area of the element. The change in resistance due to applied stress is a function of geometric and resistivity changes, which is given by the following (Johns, 2006):

$$\frac{\Delta R}{R}=\frac{\Delta L}{L}\left(1+2v\right)+\;\frac{\Delta \rho }{\rho },$$

where v is Poisson’s ratio. In metallic conductors, i.e., strain gauges, the change in resistance is mainly a function of the physical dimensions and geometric effects, $\frac{\Delta L}{L}\left(1+2v\right)$ . In semiconductors, however, the change in the bulk resistivity, $\frac{\Delta \rho }{\rho }$ , gives even a more significant contribution to the change of resistance.

Two electrodes connected to the piezoresistive element’s ends allow this change in resistance to be measured. Under loading, the induced change in the sensor's resistance can be measured by applying a constant voltage, V, and monitoring the change in the electrical current, I, or vice versa, according to the equation: ΔR = Vconst/ΔI or ΔR = ΔV/Iconst. The measured electrical change can indicate the extent to which resistance has been altered (Tiwana et al., 2012). The correlation between applied force and measured resistance (usually found to be linear) can then be calculated and implemented, usually into biomedical or automotive applications (Samaun et al., 1971; Ruhanen et al., 2008).

There are several benefits associated with piezoresistive force sensors. Due to its maturity in the market, serving as one of the most popular tactile sensing technologies in the mechanical and robotic industry, it has been widely improved and developed since its inception (Stassi et al., 2014). This has made the sensor sufficiently simple and of low cost to produce, along with improvements in making for a relatively low-power-consuming sensor (Stassi et al., 2014). Piezoresistive sensors, with their flexible physical qualities, have also been known to be less susceptible to noise and less vulnerable to shock, vibration, and temperature (Tiwana et al., 2012). High sensitivity, repeatability, and spatial resolution are also qualities that make the sensor ideal for several applications (Samaun et al., 1971). The ability to shape, flex, stretch, and scale these sensors onto gloves and skin for medical purposes also makes such a technology versatile (Sorab et al., 1988; Papakostas et al., 2002; Latessa et al., 2009; Büscher et al., 2015). Geometric scaling is known to be significantly easier compared to its counterparts (Weinstein and Bhave, 2008). Moreover, piezoresistive sensors have been used in many different biomedical applications such as intracranial pressure monitoring (Lalkov and Qasaimeh, 2017), catheters (Meena et al., 2017), and personal healthcare (Trung and Lee, 2016).

On the other hand, drawbacks include the trade-off of flexibility and sensitivity upon miniaturization (Yousef et al., 2011). For example, a decrease in piezoresistive layer thickness corresponds to an increase in both sensitivity and noise (Wang et al., 2005). The stiffness and fragility have been overcome by embedding piezoresistive sensors with flexible polymers (such as polyimides), making their conformation onto surfaces easier (Yousef et al., 2011). Piezoresistive sensors are also prone to hysteresis, an error inflicted as a result of the continuous bending or pressuring of the piezoresistive material, which may lead to retardation or temporary inaccuracies in measurement. Chuan and Chen (2011) demonstrated that hysteresis of silicon piezoresistive sensors, for instance, can be compensated through utilizing an inverse general Preisach model. The wiping-out property was found to be effective in compensating for hysteresis error and therefore proved to be a suitable solution to this piezoresistive limitation.

The most common method of integration of piezoresistive sensors for MIS is through a laparoscopic grasper. Basically, piezoresistive sensors can be mounted onto the interior surface of the jaws, where grasping force and pressure data are obtained and sent to systems to provide feedback to surgeons. Whereas the form in which feedback is provided can vary from visual and auditory to heat and vibration, this section will discuss the use of piezoresistive sensors within MIS and the conclusions such studies have garnered.

Such a proof of concept that demonstrates the suitability of piezoresistive sensors within laparoscopic graspers in robotic surgery was presented by Sarmah and Gulhane (2010). Commercially available piezoresistive force sensors were purchased from Tekscan, providing researchers with a thin-profile (208 µm), miniature (10 mm diameter), and flexible FlexiForce piezoresistive sensor. These off-the-shelf sensors were chosen for their static and dynamic performance, high linearity, and force range (0–110 N), meeting the force range standards for palpation and other laparoscopic functions as described earlier. Electrodes were composed of silver conductive strips, and researchers used inverting amplification circuits to measure force through measured changes in resistance. When coupled with a strain-gauge sensor, the sideways manipulation force was also detected, allowing test surgeons to grasp tissue through the robotic arm and simultaneously feel varying grasping pressure levels through the robotic controls. Similarly, King et al. (2009b) took this concept of directly integrating commercially available sensors into laparoscopic tools one step further, developing a functional da Vinci robotic surgical system with tactile features. By integrating the piezoresistive substrates onto the system’s cadière grasper tool, along with a multielement tactile feedback (MTF) system, they were able to prove the feasibility of the feat into commercial surgical systems. Perceptual tests with human participants showed the static and dynamic force accuracy of such an application.

**FIGURE 2:**
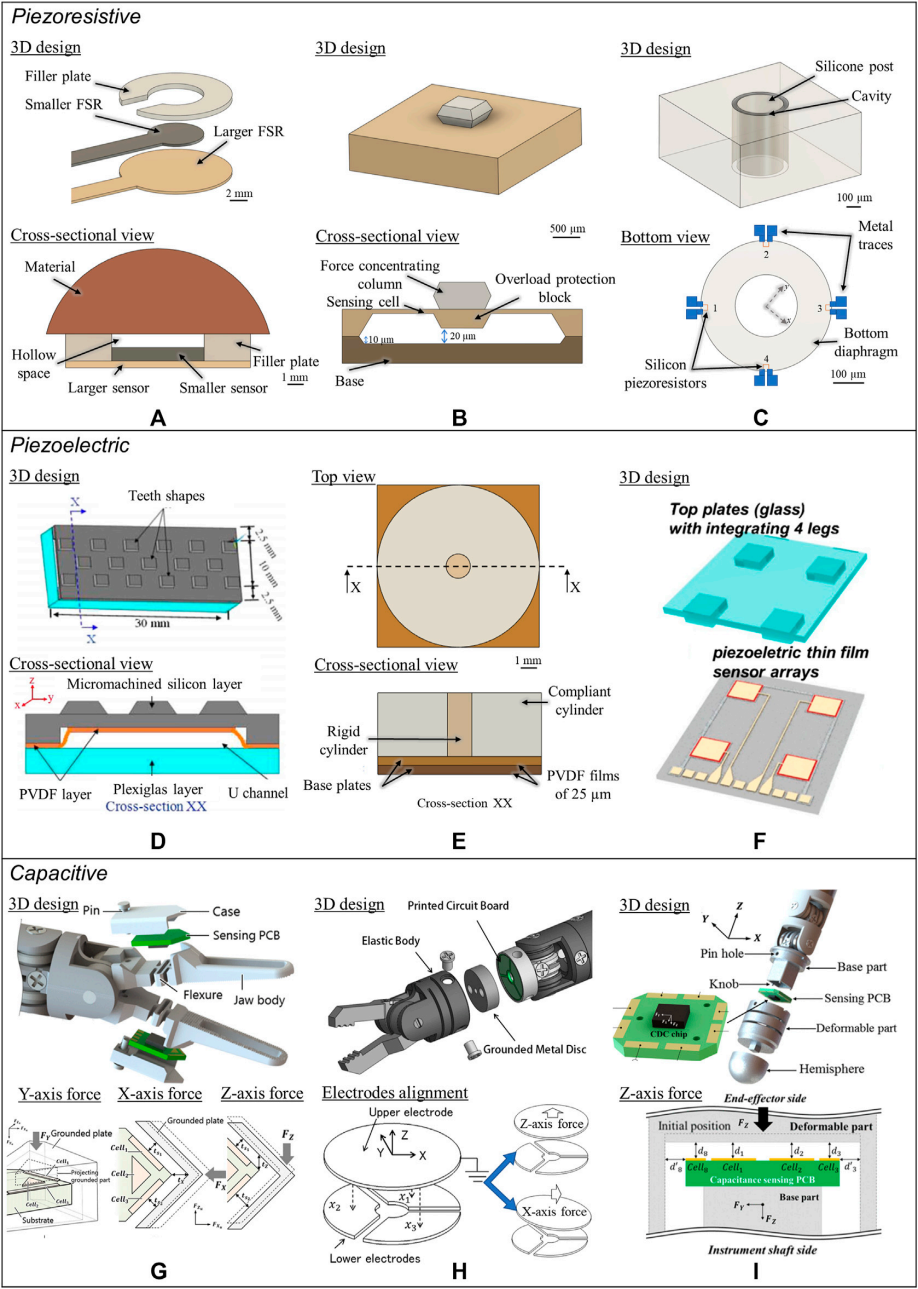
Electrical-based tactile sensors for MIS. **(A–C)** Piezoresistive tactile sensing. **(A)** Schematic and cross-sectional view of the piezoelectric tactile sensor consisting of two circular force sensing resistors with different diameters and one plastic filler plate, which can differentiate between the hardness of other types of elastomers (Kalantari et al., 2010). **(B)** Illustration of mechanical structure and a cross-sectional view for the individual three-dimensional force sensing cell of the tactile sensor (Mei et al., 2000). **(C)** 3D illustration and backside view of the bioinspired piezoelectric tactile sensor consisting of a silicone post on top of a bottom diaphragm with strain gauges (Hu et al., 2010). **(D–F)** Piezoelectric tactile sensing. **(D)** Schematic showing the structure and cross-sectional view of PVDF-based piezoelectric tactile sensor composed of three layers: a double-sided micromachined silicon plate, a PVDF film, and a Plexiglas support layer (Qasaimeh et al., 2008a). ^©^ 2008 IEEE. Reprinted, with permission. **(E)** The structural design of a PVDF-based sensor consisting of a rigid cylinder surrounded by a compliant cylinder for compliance measurement (Sedaghati et al., 2005). **(F)** Illustration of the triaxial tactile sensor employing an array of four active piezoelectric sensors (Lee et al., 2014). Copyright (2019) MDPI. **(G–I)** Capacitive tactile sensing. **(G)** Exploded view of the sensorized surgical forceps and the configuration of the 3-DOF capacitive force sensor under different applied forces showing the displacement between the sensing cells and the grounded part (Kim et al., 2018a). ^©^ 2018 IEEE. Reprinted, with permission. **(H)** Exploded view of the wrist force sensor and the electrode alignment of the three-axis capacitive force sensor at rest and under two applied forces (Lee et al., 2016). ^©^ 2016 IEEE. Reprinted, with permission. **(I)** Exploded view of the surgical palpation probe with the capacitance sensing PCB configuration and one case in which change in capacitance is generated by the capacitance sensing cells when *Z*-axis force is applied (Kim et al., 2018b). ^©^ 2018 IEEE. Reprinted, with permission.

One of the most lauded qualities of the piezoresistive sensor is the flexibility to design a sensor to fit the specific dimensions, resolution, and ranges of the sensor to serve a researcher’s purpose. Such a challenge to design, fabricate, and evaluate a piezoresistive hardness sensor for MIS was explored by Kalantari et al. (2010). Piezoresistive sensors were chosen for their quick response time, low noise in results, and ease of microfabrication. As shown in Figure 2A, the final design consisted of two piezoresistive sensors, a large (15 mm diameter) sensor capped with a ring-shaped filler plate of similar dimensions and a small (6 mm diameter) sensor embedded within the empty interior hole of the filler plate. Forces applied on the filler plate are first recorded on the large sensor. When the examined material is displaced enough to come in contact with the smaller sensor within the filler plate, the force is recorded again on the large sensor. This means that, relative to harder materials, softer materials come in contact with the smaller sensor through less applied force, implying that they are more flexible and bendable. Therefore, such a design allows the determination of a material’s resistivity for bending within the filler plate, and thus, its hardness.

Furthermore, the proposed design entails no moving parts, eliminating the need for more complex machinery that may lead to reliability issues. In an experiment to validate the sensing function in identifying the relative hardness of 6 different elastomer samples, the piezoresistive design could differentiate the hardness of three samples. However, it failed to distinguish the relative hardness of three other samples of relatively similar hardness. This proved the design’s reliability in identifying materials with considerable hardness differences, making it suitable for palpation for more pronounced tumors but unsuitable for more hidden or obscure ones.

Atieh et al. (2011) attempted to make a piezoresistive-based multifunctional sensor that could simultaneously measure the contact forces of the grasper tool, as well as the relative hardness of the material. Relative hardness was measured when an abnormally concentrated load was detected by the sensor, indicating a physically affected tissue with the possibility of a tumor. Silicone rubber samples of varying hardness were used to simulate human tissues, proving the potential of the sensor to distinguish a range of silicone rubber samples.

Three-dimensional force sensors have also been developed and fabricated by silicon MEMS technology for compactness. Mei et al. (2000) developed a compact, yet robust, piezoresistive sensing system with a soft contact surface capable of measuring up to 50 N of force. Force concentrating silicon columns positioned on the piezoresistive sensing cells were enclosed within rubber surfaces to absorb shock for the inner devices and circuitry (Figure 2B). An asymmetric strain distribution on the membrane of the 4 × 8 sensing cells was key to detecting three-dimensional forces. The design was optimized to measure forces from all directions and stresses on all zones of the sensor. Calibration to 0.5% error was performed through weight loading, and further steps were taken to ensure force detection in the X, Y, and Z directions. For 0–50 N of forces on the *Z*-axis and -10–10 N of forces on the *X*- and *Y*-axes, the final sensor achieved a 2% full-scale accuracy, indicating reasonable force detection in all three dimensions. Integration onto laparoscopic tools for MIS is yet to be determined, but such a study poses the possibility of measuring shear forces with high accuracy within MIS-acceptable force ranges through piezoresistive sensors.

While one of the prominent ways in which surgeons can judge whether tissue has a tumor is through MIS palpation with a laparoscopic grasper, Kattavenos et al. (2004) took a different approach to this medical practice by developing a sweeping force sensor. In this study, sensors were composed of a one-dimensional array of eight resistors (measuring 10 × 40 mm^2^), each with an individual sensing area (0.5 × 0.5 mm^2^). The sensor was secured onto a forceps jaw, where it swept across a phantom bowel several times in order to build a visualization of where irregular lumps of varying intensities, sizes, and gaps were. This sweeping-for-detection technique, as opposed to the grasping-for-detection, proved its feasibility in experimentation, but it required several “sweeps” in order to build a full image of possible tumor locations on the tissue. A similar feat was demonstrated by Naidu et al. (2016), who developed a low-cost disposable palpation tactile sensing device using piezoresistive sensors covering a 36 × 10 mm^2^ sensing area. The study focused on uncovering tumors of sizes smaller than 25 mm because larger tumors are known to be easily detectable without tactile sensation aids. The piezoresistive array required the sensing area to span the diameter of a tumor and come in contact with healthy surrounding tissues. An algorithm was implemented to detect whether there was an abnormal area of force detected on the sensor relative to its environment, indicating the possibility of a tumor. Detected pressure differences of above 40% of the maximum average pressure setting were indicated by red regions on the computer-simulated image.

Further testing was done to ensure that the cyclic loading on the sensor and stress relaxation induced errors did not significantly affect data, with the assumption of 0.3 Hz to be the maximum palpation cyclic load pattern with a 45 N maximum palpation force. Static loading of maximum forces over 15 s on the sensor showed a 16% drift on the piezoresistive sensors, suggesting that improvements need to be made to compensate for stress relaxation effects. Phantom palpation tests were performed, with silicone rubber spheres simulating tumors five times stiffer than healthy tissues, placed at different depths to examine sensor sensitivity. The system was able to detect a simulated tumor up to 10 mm deep clearly. Furthermore, tissue palpation tests were performed with six novice subjects (pre-trained with instrument operation) to locate randomly hidden tumors on a bovine liver sample. All subjects successfully reported all tumors without false positives, with a localization error of 2.2 ± 0.9 mm. The study demonstrated that the system was user-friendly for novices without MIS training. Such systems may prove useful in cases where surgeons cannot directly grasp a visible tumor or irregular tissue, but the research falls short of experimenting with excised or diseased human tissue, where detection may prove to be a greater challenge.

Taking on a more unique approach to developing a piezoresistive-based sensor for MIS, Hu et al. (2010) developed a bioinspired tactile sensor mimicking the structure and mechanism of the hair cell. Inspired by the hair cell’s ability to detect mechanical stimuli with great sensitivity and durability, the team tested a tactile sensor composed of a central silicon post, surrounded by four piezoresistive sensors (acting as strain gauges) fabricated on a thin-film polyimide diaphragm base (Figure 2C). Due to mechanical stimuli, the tilting of the central silicon post led to deformation on the diaphragm base, which was measured by the four piezoresistive sensors. Excessive shear stimuli (bending) were resolved by building a cylindrical wall, limiting the central posts’ displacement. A balanced trade-off was made between building a high central silicon post with high sensitivity but poor durability. The final fabricated sensor measured 3.5 N^−1^ and 10.8 N^−1^ of normal and shear force sensitivities. The force experienced by the central post is usually much smaller than that of the total force applied on the entire sensor, making the sensor suitable for MIS. The sensor was able to detect a minimum of 0.046 and 0.017 mN of normal and shear forces, making it capable of detecting minimal force changes. However, the shear force measurement was limited to a 0.05 N maximum. Finally, a scratching test was performed using rubber fingers to prove that data on scratching patterns (direction, speed, and intensity) could be recorded by an array of sensors. With high-sensitivity normal and shear forces detection, the sensor was ideal for 3D force applications. Although promising in terms of specifications, more work is required to process signals from the sensor on-chip (within the MIS tool) in order to prevent wires from interfering with MIS. Integration onto a laparoscopic instrument can then be made possible, and the feasibility of the system fully demonstrated and optimized for MIS.

Realizing the importance of protecting electronic circuits of the force sensor from the surgical environment (with organs, bodily fluids, blood), as well as preventing the contamination and infection of a patient from the laparoscopic tool in MIS, Radó et al. (2018) focused on piezoresistive sensor development on creating an appropriate elastomer coating. Polydimethylsiloxane (PDMS) coating was selected for its elastic and sterilizable properties. The PDMS coating of a perpendicular load sensor led to an increased sensitivity deviation of from ±3% to ±10%, with a delay in the response time from 36 to 60 µs. It is suspected that the tendency of PDMS to conform while deforming, as well as elastomer hardening across certain regions, led to these deviations. The system was later attached to a Robin Heart surgery robot, but the study did not indicate specific tests or experimental results performed.

As one of the most mature and developed mechanical sensing technologies, piezoresistive force sensors are low-cost, have shape versatility, and can reliably produce force data. They are relatively flexible, durable, and consume relatively little power. However, issues such as increased noise and decreased sensitivity arise when these force sensors are miniaturized (Wang et al., 2005). Smaller, thinner piezoresistive sensors are also more prone to fragility and hysteresis error. Despite this, researchers were able to show that this technology is feasible in commercial surgical systems through implementing piezoresistive tactile sensors in surgical graspers. For example, Atieh et al. (2011) suggested that these sensors could detect a range of tissue hardness values required for palpation. Another implementation included the fabrication of a sweeping piezoresistive sensor array that was used to accurately detect tumors up to 10 mm deep without false positives on bovine liver samples (Kattavenos et al., 2004). Other non-surgical related studies have shown the ability of piezoresistive sensors to detect three-dimensional forces, which could be useful for measuring shear forces with high accuracy. Although well-studied and researched, more testing of piezoresistive force sensors in actual clinical settings is needed to determine its worthiness in MIS.

### 4.2 Piezoelectric Tactile Sensing

Piezoelectric force sensors rely on the piezoelectric effect, wherein imposed mechanical forces onto a piezoelectric element lead to measurable generated charges that can be harvested as the output voltage (Tzou and Tseng, 1990). Each given force corresponds to a certain charge across the sensing elements. An amplifier converts this to an output voltage proportional to the pressure. Applications range from force and pressure detection to acceleration and vibration measurements (Damjanovic, 1998). Conversely, converse piezoelectric sensors can harvest vibrations and movements into stored energy (Roundy and Wright, 2004; Ng and Liao, 2005). The basic relationships of the direct and converse piezoelectric effects can be described by the piezoelectric constitutive equations, represented by the following equation (Tadigadapa, 2010):

$$D_{i}=d_{\mathrm{ij}}\sigma_{j}+\varepsilon_{\mathrm{ii}}^{T}E_{i}\;\mathrm{or}\;D_{i}=e_{\mathrm{ij}}S_{j}+\varepsilon_{\mathrm{ii}}^{S}E_{i},$$

and

$$S_{j}=s_{\mathrm{ij}}^{E}\sigma_{j}+d_{\mathrm{ij}}E_{i}\;\mathrm{or}\;T_{i}=c_{\mathrm{ij}}^{E}S_{j}-e_{\mathrm{ij}}E_{i},$$

where $D_{i}$ is electrical displacement, $\sigma_{j}$ is the mechanical stress, $\varepsilon_{ii}$ is the permittivity, $E_{i}$ is the electric field, $S_{j}$ is the mechanical strain, $T_{i}$ is the Temperature, $s_{ij}$ is the elastic compliance coefficient, and $c_{ij}$ is the elastic stiffness constant. The superscript on one parameter indicates when another parameter is held constant, such as $s_{ij}^{E}$ , which represents the elastic compliance coefficient under a constant electric field. The piezoelectric coefficients $\;d_{ij}$ and $e_{ij}$ correspond to a 3 × 6 matrix, where the indices (*i* = 1–3) define the normal electric field or displacement orientation, (*j* = 1–3) define normal mechanical stresses or strains, and (*j* = 4–6) represent shear strains or stresses. Another important figure of merit in piezoelectric materials is the electromechanical coupling coefficient, *k*, representing the ratio of the mechanical (electrical) energy converted to the input electrical (mechanical) energy for the piezoelectric material. The coupling coefficient is the square root of the following equation (Tadigadapa and Mateti, 2009):

$$k_{33}^{2}=\frac{d_{33}^{2}}{\varepsilon_{33}^{T}S_{33}^{E}}.$$

Commonly found piezoelectric elements include mainly natural and human-made crystals (quartz, salts, and topaz) or ceramics (Jaffe and Berlincourt, 1965; Damjanovic, 1998). The piezoelectric material holds certain axes of polarity, allowing the propagation of the piezoelectric effect (Gallego-Juarez, 1989). These crystal properties, including its lattice structure and cut shape, allow for generating of voltage potentials that can distinguish normal, longitudinal, and shear forces (Tiwana et al., 2012). When the piezoelectric element is deformed by applied pressure, the induced polariztation and, subsequently, generated voltage are directly proportional but decay through time dictated by the material’s dielectric constant and impedance (Gautschi, 2002; Tiwana et al., 2012). This makes such a sensor design ideal for dynamic forces (especially at high-frequency) but renders it ineffectual when measuring static forces over an extended period of time.

In fact, the use of the piezoelectric effect has matured within medicine in the past three decades through a field called piezoelectric surgery (Labanca et al., 2008). Piezoelectric surgery utilizes vibrational ultrasonic frequencies to cut through hard tissues while keeping soft tissue intact (Siervo et al., 2004; Schaller et al., 2005). This minimally invasive technique lowers risks associated with oral and maxillofacial surgeries, making it one of piezoelectricity’s most impactful contributions (Labanca et al., 2008).

The main advantages of piezoelectric sensors include high stability (when single crystals are used), reproducibility, and linearity (Gautschi, 2002). Its frequency can range from 1 Hz to the MHz level, allowing it to detect high-frequency motions (ideal for vibrations). Such sensors possess one of the highest span-to-threshold ratios (over 10^8^), allowing a great measuring range from mN up to kN (Tressler et al., 1998). Due to the piezoelectric material’s composition, the sensor is also rendered mostly unaffected by changing electric and magnetic fields in the surgical environment (Tressler et al., 1998; Gautschi, 2002). Their ability to be compacted and embedded within health monitoring systems makes them ideal for medical implementation (Sirohi and Chopra, 2000). Likewise, complex shapes and large areas can be easily realized (Tressler et al., 1998).

The major drawback of the piezoelectric force sensor is, as discussed earlier, its inability to measure static forces over long periods of time. To measure static forces over a long duration using piezoelectric properties, perfectly insulating materials and near-zero internal resistance are needed to prevent the constant electron loss in the sensor. Partially static measurements are made possible using a single crystal as the piezoelectric medium (Gautschi, 2002). Water-soluble crystals used in piezoelectric sensors may also become susceptible to highly humid environments. Charges from the surrounding environment (if the piezoelectric material is exposed) may affect measurements as well. Its temperature sensitivity may also lead to inaccurate measurements and crystal deformation due to the thermal expansion and temperature-dependent properties of the pyroelectric, piezoelectric materials (Zhang and Yu, 2011). Fortunately, its temperature sensitivity can be disregarded in MIS environments.

Polyvinylidene fluoride (PVDF) is one of the most widely applied piezoelectric elements used in force sensing integration in MIS due to its ability to be manufactured into thin sheets (Puangmali et al., 2008). Dargahi et al. (1999) reported a microfabricated tactile sensor for MIS that can both detect the magnitude and location of applied forces on a commercially available laparoscopic grasper. Results showed that the sensor had high linearity and decent sensitivity of 0.1 N. However, to avoid damage to the sensor, the maximum tested load was 2 N per sensing element. MIS forces can reach up to 35 N, so ideally, sensing elements should have a greater range. Although the developed PVDF-based tactile sensor could be attached without altering the original laparoscopic grasper, the design was proven to be complicated and cumbersome and, therefore, unsuitable for easy commercial integration.

Miniaturized specifically for MIS integration, a multifunctional PVDF-based tactile sensor was made by Sokhanvar et al. (2007). A total of three PVDF sensing elements were implemented onto the tissue grasper, two of which were attached on the ends of a flexible beam to determine force magnitude and position, while a third sensing element was attached to the center of the beam to measure material hardness. Softness characterization was made calculable through the values read from the two end sensors and the deflection/stress induced on the center sensor. Softer grasped materials led to larger beam deflections. The sensor was validated both analytically and numerically, and it was indicated that the results were satisfactory with theoretical data with high sensitivity and MIS-appropriate range. It should be noted that the results also indicated that a trade-off between the range of stiffness and resolution had to be made. To achieve an ideal balance between sensitivity and resolution, properties of the flexible beam (material, length, and thickness) would have to be altered according to the specific surgery. In addition, only dynamics loads were tested. Further work is needed to be done to micromachine the sensor and test it with more complex soft tissues to analyze the device-tissue friction.

Qasaimeh et al. (2008a) advanced the concept further by improving the design and miniaturizing it using MEMS technologies. The team developed a fully micromachined PVDF-based sensor accommodating the full range of forces associated with MIS (Figure 2D). In the jaw design, a patterned PVDF film was sandwiched between a micromachined silicon layer with tooth-shaped protrusions and a Plexiglas layer. A 200 µm gap between the PVDF film and Plexiglas layer was made in order to allow for the silicon plate to deflect upon object contact. Upon contact, plate deflection stretched the attached PVDF, providing voltage output and, subsequently, force readings. Three sensing units, each composed of two sensing elements at the silicon plate supports and one on the silicon plate bridge, made up the complete sensor. This design allowed the sensor to measure both magnitude and relative position on the contact force on the sensor. Simulations were carried out, showing that the sensor was able to detect hidden irregularities within a grasped object. Sudden changes in force or uneven uniformity of measured force indicated the presence of lumps. Softness estimations of different elastomers were carried out using the microfabricated sensors, with the observation that a higher grasping force leads to a smaller deviation between the theoretical and experimental calculation of the modulus of elasticity (Qasaimeh et al., 2008b). The sensorized grasper jaw also exhibited the ability to detect small forces from simulated pulsating arteries (assumed to be dynamic, with few grams of force) while measuring large grasping forces. Moreover, since it was micromachined, it could be mass-produced with a low unit cost and be disposable (Qasaimeh et al., 2009). More realistic testing is needed to determine its ability to detect hidden tumors and other unexposed tissue features, perhaps with animal tissues.

In a study by Chuang et al. (2013), a novel approach of using a small steel ball embedded within a soft material allowed for a flexible tactile sensor for piezoelectric-based MIS. In the study, a PVDF film detected different physical properties of objects by determining uneven stress distributions from the applied force due to the stiffness difference between the steel ball and PDMS. Such a sensor was used to characterize different soft tissues of animal organs by hardness through cyclic loading of the material. For softness estimation of gripped organs using a smart MIS grasper, a further comparison of the obtained results with an experimentally generated database of each organ and tissue is worth every effort (Azizi et al., 2018). Ultimately, recognizing the tissue and the maximum force that the surgeon can apply on it is possible.

In a proof-of-concept study, Sedaghati et al. (2005) took a different approach to determine the compliance of tissues in MIS through a cylindrical PVDF design. Two cylinders were used: a phenolic rigid cylinder was wrapped with a larger, soft rubber deformable cylinder (Figure 2E). A PVDF film between the rigid cylinder and base plate was used to capture the forces experienced by the rigid cylinder. Another PVDF was placed between the two rigid Plexiglas plates (beneath the two cylinders) to measure the total applied force. The prototype and experiment proved that by determining the ratio of the force applied onto the rigid cylinder to the total measured force on the sensor, the softness of the object could be determined. Good agreement was found between the tested results and finite element results. Although the current prototype exhibits high sensitivity and linearity, miniaturization has not been proven yet, rendering the current sensor design unsuitable for MIS integration. Sedaghati et al. (2005) acknowledged this drawback and noted that a miniaturized sensor may face accuracy concerns and could be damaged by large shear forces. Further investigation regarding this piezoelectric force sensor design is needed.

A similar design of using rigid and compliant cylindrical bodies to determine the viscoelastic characteristics of tissues was studied by Narayanan et al. (2006). As opposed to previous piezoelectric-based sensors studied by Dargahi et al. (1999), Dargahi (2002), and Dario et al. (1984), which measured tissue compliance exclusively, the proposed sensor was designed to measure both compliance and viscous damping in tissues. Because tissues are viscoelastic, Narayanan et al. (2006) deemed the development of a sensor capable of determining viscosity as important for improved tissue characterization and modeling. Testing was performed to verify the sensor. It was found that rapid loading and unloading cycles of the target material were required to determine the viscoelastic properties of the material. Because the ability to find the viscoelastic property increases with the increased loading rate, real-life palpation would require fast grasps on different parts of tissue in order to determine viscosity. Although the concept is proven, such a system would be impractical in its current state if mounted onto endoscopic or laparoscopic graspers in surgery.

Meanwhile, several researchers particularly attempted to measure forces applied by catheters and endoscopes, as these instruments are commonly used in MIS. In Chuang et al. (2016)’s work, a miniaturized tactile sensor was made suitable for mounting on the tip of an endoscope to detect submucosal tumors by hardness assessment. This PVDF-based piezoelectric tactile sensor involved a copper ball embedded in soft packaging, where the voltage ratio obtained from the hard inner ball and soft packaging layers indicated the hardness of the contacted object. They claimed that this sensor is safe to be used for actual endoscopy due to the passive nature of the sensing element, as well as using the biocompatible PDMS for packaging.

Another innovative approach towards integrating PVDF piezoelectric tactile sensors into miniaturized systems was explored by Li et al. (2008)., in which a high-sensitivity dome-shaped flexible sensor was fabricated and tested. The study presented a novel “mold-transfer method” to producing piezoelectric polymer films that could easily be fabricated to conform to any given shape, making it ideal for a wide range of biomedical applications. The micromachined mold, which matched the shape of the desired application surface, was formed, spin-coated, applied with the piezoelectric polymer solution, and then integrated onto the actual device itself. For a bump-shaped design, polyvinylidene fluoride-trifluoroethylene (PVDF-TrFE) was used for the polymer solution, and SU-8 was used for the bump mold. For fabrication of the dome-shaped film, PVDF-TrFE solution was spin-coated onto cyclic-olefin-copolymer lens molds. A protective layer of parylene film acted as a thermal isolator to avoid temperature and pressure variations associated with ferroelectric materials. Dynamic forces at 5 Hz were successfully tested with the sensor for loading ranges between 20 mN and 1 N. Force increments of 40 mN (for the bump-shaped sensor) and 25 mN (for the dome-shaped sensor) could be measured. The study illustrated an easier way to fabricate miniaturized biomedical tactile sensors and proved its high-sensitivity capabilities for simple force measurements. Forces encountered within MIS are often more complicated, so further developments are needed to give the fabricated sensors the ability to determine the shape, location, and hardness of tissues. Direct applications such as tissue palpation and tissue property detection were not discussed.

Outside of the more popularly used PVDF, Lee et al. (2014) selected piezoelectric polycrystalline lead zirconate titanate (PZT) for enhanced sensitivity when integrated with a micro-structured PDMS element. The proposed structure was composed of a top glass plate with four stress-concentrating columns with the PZT sensor layer as the base (Figure 2F). The four individual piezoelectric force sensors below the glass columns allowed both force direction (shear) and location to be easily measured. Moving forces could be detected, and the design was miniaturized with MEMS technology, allowing the simple sensor to be applied for biomedical tactile applications. Although the proof of concept was complete, actual experimentation with LS or palpation was not tested, suggesting that more work is needed to determine its usefulness in either practice in detecting hidden lumps or irregular tissues.

Ottermo et al. (2004) also selected PZT piezoelectric sensors for their ability to be easily miniaturized into an array of 30 sensing elements (3 × 10) to measure forces and their locations. The described work was intended for an augmented MIS, in which a tactile display installed onto the physician’s finger would reflect forces and shapes grasped by the tissue. However, such a design lacked the ability to tell tissue hardness, and the tactile feedback was not proven useful to physicians. Issues encountered during the prototyping phase included the narrowness of the sensing area and challenging integration with the tactile display. Such a proposed concept has yet to be created and tested.

Other advanced piezoelectric sensors were designed in a spiral-shaped structure for the estimation of tissue hardness using catheters. For instance, the sensor developed by Zhang et al. (2017) consisted of a square spiral metal plate designed to reduce the sensor’s resonant frequency and, therefore, restrict the impact brought by the effective mass of the tissue. The number of the sensor’s components was reduced using one ceramic of PZT as both an actuator and a sensing element. The detection of a lump inside a silicone sample was demonstrated after successfully verifying the sensor’s ability to measure hardness. A further miniaturized and optimized sensor was presented later, having a circular sensing element of a spiral shape with an outer diameter less than 8 mm, which was integrated on the tip of a catheter (Ju et al., 2019). However, one drawback was the change in the sensor’s sensitivity with the change in the hardness of the tested samples. This type of measurement is classified as frequency-domain tactile sensing.

Piezoelectric sensors offer many important advantages, including stability, reproducibility, and linearity, that make them suitable for many force detection operations. The sensor can be easily compacted and is unaffected by changing electric and magnetic fields, making it ideal for medical implementations (Sirohi and Chopra, 2000). Although they can be temperature sensitive, the range of piezoelectric thermal expansion associated with MIS is not significant enough to lead to inaccurate measurements. A drawback of piezoelectric tactile sensing that also deserves attention is its inability to measure static forces over extended periods of time (Gautschi, 2002). Despite this, PVDF has been widely used for MIS grasper integration in research with considerable success. Qasaimeh et al. (2008a) fabricated a PVDF-based jaw sensor that was able to detect small, hidden irregularities in objects but fell short of actual clinic testing. Li et al. (2008) proved that PVDF sensors could be miniaturized and molded into different shapes while preserving their high-sensitivity capabilities for simple force detecting tasks. Many similar studies worked on placing these sensors into arrays or different shapes but did not do much in actual clinical testing. Advanced and miniaturized systems, e.g., piezoelectric needle sensor, can be useful for tissue diagnosis by revealing the biomechanical variations of tissues caused by lesions, e.g., human thyroid (Sharma et al., 2019). For piezoelectric tactile sensing to play a major role in LS in the future, researchers need to further prove its versatility and efficacy in detecting tumors and more complicated shapes in clinical settings.

### 4.3 Capacitive Tactile Sensing

Capacitive sensing has acquired an extensive interest in circuit design for its high electrical sensitivity, excellent repeatability, low power consumption, compact layout, linear response, simple device construction, and immunity to temperature variation and thermal noises, in comparison to its piezoelectric and piezoresistive counterparts (Zhou et al., 2005; Chi et al., 2018). Recently, capacitive sensors were introduced to a wide range of biomedical applications, such as bio-analytical detectors (Wongkittisuksa et al., 2011), smart implants (Iqbal et al., 2019), prosthetic skins (Mannsfeld et al., 2010), and wearable electronics (Pan and Wang, 2011). Typically, capacitive sensors consist of pairs of electrodes separated by a dielectric medium. The value of electrical capacitance, C , of parallel-plate capacitor can be calculated by the following simple, well-known governing equation:

$$C=\frac{\varepsilon_{0}\varepsilon_{r}A}{d},$$

where $\varepsilon_{0}=8.85\times 10^{12}\;F/m$ is the vacuum permittivity, $\varepsilon_{r}$ is the relative permittivity, A is the area of electrodes, and d is the distance between electrodes. The relationship between the magnitude of the applied normal force, F , on the two parallel plates and the output voltage, V , of the capacitor is written as follows (Bao and Bao, 2000):

$$F=\frac{\varepsilon_{0}\varepsilon_{r}A}{2\;d^{2}}V^{2}=\frac{C}{2d}V^{2}.$$

In capacitive tactile sensors, the applied mechanical loading, e.g., pressing or stretching, compresses the spring-like dielectric material and changes the effective area of and the distance between the two electrodes of the capacitor. Therefore, the dielectric layer is designed to be highly deformable, allowing the capacitive sensor to be responsive to minimal compressive loadings. In particular cases, the dielectric properties of the medium separating the electrodes can be changed by an external load, i.e., forcing another material of different permittivity into the sensing element. Eventually, the capacitance of the sensor will be altered. Circuitry translates the measured capacitance change into force differential and retrieves the mechanical signal. Basically, the measurement range and sensitivity of the sensor can be adjusted by changing the compliance of the dielectric material, e.g., PDMS elastomers with different mixing ratios (Lei et al., 2014).

In one demonstration, a flexible, capacitive tactile sensor array was developed using PDMS as a base material with the capability of measuring both normal and shear force distributions,(Lee et al., 2008). The design of each tactile cell incorporated a large bump on top of a pillar structure formed at the center between the air gap of four capacitors. Applied normal forces induced an equal capacitance change across all capacitors, whereas shear forces corresponded to a capacitance increase in two elements and a countereffect on the adjacent ones. The individual sensor cell within the proposed setup showed sensitivities of 2.5%, 2.9%, and 3.0%/mN in the X, Y, and Z directions, respectively.

Recent advances in photolithography techniques can enhance the spatial resolution and reduce the overall thickness of capacitive sensors by miniaturizing the sensing elements and eliminating the need for adhesive layers between capacitive plates (Pritchard et al., 2008). Additionally, the performance of capacitive tactile sensors can be improved by developing well-designed electrodes. High-performance, flexible capacitive tactile sensors were achieved using a bottom micropatterned elastomeric electrode fabricated by coating ultrathin sliver-nanowires (AgNWs) onto the PDMS layer with uniform microtower patterns (Wan et al., 2018). The high aspect ratio and low density of the micropatterns make them easier to deform than solid dielectric films, leading to an increased pressure sensitivity of 1.2 kPa^−1^.

Several capacitive tactile sensor arrays have been mounted on MIS graspers to measure the exerted force by the surgeon during procedures. In Ottermo et al. (2006)’s work, an array of 15 × 4 thin capacitive pressure sensing elements was fixed onto a grasper jaw that offered the detection of pressure distribution in a range up to 7 N/cm^2^ with 2 mm spatial resolution. While conventional graspers usually have a serrated surface, the smooth surface of the proposed sensor array escalated slippage occasions and created more problems for the inexperienced subjects. Nevertheless, delivering visual feedback of the tactile image was totally helpful for discriminating between objects of different hardness and sizes. Towards satisfying both compatibility and electrical constraints of clinical implementation, Paydar et al. (2012) fabricated a capacitive sensing device with a material choice of parylene C and gold for the insulating dielectric medium and capacitive sensing plates, respectively. MEMS processes of lithography and chemical vapor deposition were employed to fabricate the thin-film capacitive sensors, providing a miniaturized, low-profile, biocompatible solution for measuring forces as a basic component of tactile feedback systems for MIS.

The shape and functionality of MIS graspers can be preserved by integrating the sensor underneath the surface of the jaws. Kim et al. (2014) proposed restoring the tactile sensation via a pair of dual axial force sensors. Each sensor was made of two capacitive sensor units adhered to the surfaces of a triangular prism portion of the jaw. Here, the upper electrode plates of the sensing units were designed to be larger than the bottom electrodes to eliminate the nonlinearity in capacitance change concerning the electrodes’ overlapped area. Each jaw with a single sensor can extract force measurements along the normal and one longitudinal direction out of the differential signal of the capacitive sensing units. With the reading from two orthogonally oriented sensors, the forceps can estimate a 3-axial pulling force and a single axial grasping force. Thorough analyses of the sensing principle and the force transformation method were addressed (Kim et al., 2015), showing errors of 0.1 N with good repeatability and low hysteresis. Performance verification of the proposed sensing system consisting of a four-axial joint, tool shaft, joint actuation unit, and sensorized forceps was carried out using Raven-II, an open-source surgical robot platform. Pulling and grasping forces were estimated based on the measured cell forces, and the transformations closely matched that of a reference sensor with slightly higher noise. Yet, the proposed design focuses on tissue handling with the front portion of the inner surface of the sensorized forceps.

With the aim of measuring forces other than ones applied to the inner surface of the jaws, Kim et al. (2018a) came up with the unique idea of installing two compact 3 degrees-of-freedom (DOF) sensors at the proximal region of the forceps jaws (Figure 2G). Each capacitive-based force sensor was constructed out of orthogonal and parallel arrangements of capacitance-sensing units in a triangular structure. Using a transformation matrix with a geometric relation to the forceps, two 3-DOF forces measured by the sensorized forceps were transformed into grasping force, 3-DOF manipulating force with a palpation function, and rotational torque. The proposed sensorized forceps were taken one step further by compensating for some environmental factors, including effects of humidity, temperature, and high voltage (Seok et al., 2019). For humidity, a fourth capacitive unit was integrated within the original structure of the sensor, where its capacitance was solely influenced by humidity. As a result, the sensor can eliminate the humidity noise from the force readouts of the three other capacitive units. Since temperature influences were linked to the induced parasitic capacitance between the ground and different capacitive cells separated by the printed circuit board (PCB) layer of dielectric nature, an AC shielding layer was inserted in-between to prevent the force measurements from being affected by the change in temperature. Lastly, blocking high voltages that cause damage to the sensor was achieved by immersing the aluminum-based forceps in an acidic electrolyte to energize its surface and produce an outer layer of nonconductive aluminum oxide. Experimental results illustrated error-free grasping force under the electro-cautery process.

So far, installing sensors onto the grasping tip has been the most common way to measure the grasping force during MIS. Alternatively, the measurement of forces applied during MIS, i.e., manipulation force and grasping force, can be detected *via* sensors placed either at the wrist, shaft, or base of the tool. These positions offer a larger space for sensor placement and reduce the size constraints of the tactile sensor design. In this regard, Lee et al. (2016) presented a 4-DOF grasping tool with a miniaturized wrist force and torque sensors for tissue manipulation sensing (Figure 2H). The wrist force sensor was made up of a PCB of three discrete in-plane lower electrodes sharing a common electrically grounded metal disc as an upper electrode. For grasping force measurement, two torque sensors were embedded into the driving pulleys. Once torque is applied, the gap distance between the sensing electrodes is reduced, resulting in a measurable change in the capacitance. With both sensors, three-axis manipulation force and single-axis grasping force measurements were obtained. Subsequently, system-level validation through 1 min experiments of pulling and releasing an elastic tissue object repeatedly in arbitrary directions was performed using Raven-II. The prototype showed a well-matching response to that of a reference sensor. However, the elastic body used for assembling the wrist force sensor limits the sensing range to 1 N; hence, an enhanced design or more robust material must be considered.

Other than graspers, MIS probes have utilized capacitive tactile sensors for performance enhancements. As the early detection and removal of small pulmonary nodules could improve long-term survival rates of lung cancer patients, Miller et al. (2007) presented a capacitive tactile imaging system capable of localizing lung nodules. The system, consisting of a capacitive sensor array mounted on an MIS probe and integrated with the thoracoscopic imaging, allows the surgeon to locate hard nodules by scanning the surface of the lung and monitoring the variation in contact pressures to resolve the relative elasticity of the underlying tissue. However, the joint location and the manual control of the probe complicate the mechanical forces required to achieve good measurements. Other probes incorporating capacitive tactile sensors have been proposed for palpation in MIS as an alternative modality to using ultrasound probes. Naidu et al. (2016) introduced several novel designs of mass-producible, low-cost, sterilizable tactile sensor arrays with a 2 × 2 mm^2^ spatial resolution and a scan rate of 30 Hz, all with minimal wiring. The ability to localize 6 mm diameter and 10 mm deep tumors was shown in a silicone phantom and *ex vivo* tissue samples. Kim et al. (2018b)’s work presented another MIS palpation probe with capacitive-based force/torque sensing capability. The miniaturized sensor is composed of a deformable part, a sensing PCB, and a base part (Figure 2I). The experimental results of palpating a pig kidney and cancer simulation in a robotic surgical operation indicate the ability of the probe to recognize tumor regions and detect stiffness variance between regions. Another way to measure tissue elasticity was achieved *via* an array of capacitors of different stiffnesses, i.e., varying the sizes of sensing membranes within capacitors (Peng et al., 2009). Subsequently, the relative deflections of the sensing diaphragms correspond to the elasticity of the palpated tissues. Additionally, integrating commercially available capacitive-based pressure sensors, e.g., pressure pads, with surgical probes is one simple solution to restore the sense of touch and improve the accuracy of locating tumors in MIS (Trejos et al., 2009).

MIS tools, i.e., graspers and probes, equipped with capacitive force sensors, show great potential towards restoring tactile sensation to surgeons. Moreover, capacitive force sensors enable multi-axis tactile feedback for clinical applications of robotic surgery, which improves the speed and outcomes of procedures and leads to increased use of these robotic systems in MIS (Dai et al., 2017). Such sensors possess several advantages, e.g., ease of design and fabrication, immunity to thermal noise, and tunable spatial resolution (Puangmali et al., 2008). The capacitive sensors can be easily integrated with MEMS technology to design thinner dielectric layers. Meanwhile, the sensitivity performance of capacitive force sensors can be enhanced by integrating multiple vertically integrated sensing electrodes (Hsieh et al., 2021). Besides standard complementary metal-oxide-semiconductor (CMOS) and MEMS fabrication processes, printing technologies, such as screen printing and inkjet printing, have been adopted to manufacture flexible, thin-film capacitive tactile sensors. Printable capacitive electronics can also involve novel materials of carbon nanostructures and metallic nanowires. These extra features of printed electronics aid in enhancing the biodegradability, stretchability, or biocompatibility of capacitive-based force sensors (Rivadeneyra and López-Villanueva, 2020). Despite those advantages, the use of capacitive sensors in very high-precision applications has been limited by the compromised repeatability due to hysteresis and cross-talk. Another disadvantage of capacitive force sensing is the non-linearity due to the inversed proportionality between the output and the gap between the parallel sensing plates. While the different multi-DOF capacitive force feedback systems have been proven beneficial for preventing tissue damage caused by surgeon’s grasping and incipient slips, design limitations and surgical environment constraints are the leading causes of the delay in commercializing sensorized MIS tools.

### 4.4 Optical Tactile Sensing

Optical tactile sensing, or fiber optic tactile sensing, varies depending on design and application. This technology has been continuously evolving and used commercially for over 60 years (Krohn et al., 2014). The general concept behind a fiber optic sensor works by transmitting light through an optical fiber to a detector. The sensor modifies the light’s characteristics, such as intensity, wavelength, amplitude, phase, as a result of a change in the external environment (Krohn et al., 2014). These external changes may include pressure, strain, acceleration, temperature, electromagnetic fields, or even chemical compositions (Udd and Spillman, 2011). After the light is manipulated by the sensor in a specific manner, the modulated light travels to a signal processor, in which the qualities of the newly perceived light are compared to the original light. Based on the extent of the light’s altered characteristics, the extent of changes in the external environment, applied force in our case, can be determined. An optical fiber-based force sensor measures the applied force based on the modified qualities, such as polarization and intensity, of light sent through the optic fiber. The most commonly used fiber optic sensor is the fiber Bragg grating (FBG) sensor, in which short segments of Bragg reflectors sensors reflect particular light wavelengths while transmitting others (as a result of external changes detected) (Udd and Spillman, 2011; Krohn et al., 2014). Originally used within telecommunications, military, and aerospace, they are now being deployed in more engineering to biomedical applications (Grattan and Meggitt, 1995; Othonos, 2000). Some applications include the long-term monitoring of bridge health and safety, environment humidity sensing, or pH and blood pressure sensors in medicine (Peterson and Vurek, 1984; Li et al., 2004; Yeo et al., 2008). Despite having a broad application, for our purposes, we will mostly focus on its implementation within MIS. Deformation of the fiber due to applied strain or force can lead to measurable changes in radiation losses and decreases in transmitted light. In other designs, forces can cause polarization that changes light amplitude (Udd and Spillman, 2011).

Optical fiber sensors are well known for their versatility. Easily miniaturized, lightweight, and flexible, while remaining high in sensitivity and large in bandwidth, applications within small spaces are made possible through optical fiber sensors (Lee, 2003). However, one of its most accomplished properties that makes it suitable for MIS is its biocompatibility. Compatibility with sterilization, various chemical interferences, and electromagnetic interference make it a good candidate, considering that these are important qualities required for an appropriate MIS sensor. Fiber sensors can be multiplexed on a single network, allowing the technology to measure forces in different locations on the fiber or measure different environmental factors on the same fiber (Grattan and Sun, 2000). In addition, it is noteworthy that fiber optic sensors generally possess high resistance against strong vibrations and high temperatures. Its magnetic resonance imaging (MRI) compatibility is also a significant advantage, which will be later discussed.

There are, however, major drawbacks of fiber optic sensors that may be deal breakers for developing easily produced, affordable sensors for MIS. Fiber optic sensor systems are often more expensive than their non-optical counterparts. The systems are usually much more complex in concept, requiring a lot of precision engineering and specific installation procedures. These drawbacks, however, can be overcome through well-designed, MIS-specific sensors. Many optical fibers are also not as flexible as their electronic competitors (Ahmadi et al., 2011).

When it comes to the application of technology within MIS, optical technology may be one of the biggest contributors. Endoscopes, made from glass optical fibers, have made MIS possible by providing imaging and visual aids to surgeons. In procedures such as colonoscopy, the benefit of endoscopes to view the interior of the colon in order to remove cancerous polyps cannot be undermined (Udd and Spillman, 2011). Other optic-based technologies, such as fiber optic laser surgery, require high-power lasers to cut and remove targeted tissues.

In 1990, Hirose and Yoneda (1990) developed an optical force sensor that opened the door for optical technology deployment in robotic and medical force sensing. A transduction element modulated the light according to the applied force, which was subsequently read and interpreted by an optical detector.

Peirs et al. (2003) and Peirs et al. (2004) chose optical fibers when developing a suturing-ready minimally invasive tactile sensor for its immunity from producing leaking currents or interference. Three optical fibers were radially arranged with 120° intervals, designed to measure the displacement between the upper and lower component of the deformable sensor by measuring the intensity of reflected signals in the optical fiber. When normal forces are applied to the sensor, the three fibers will reflect the same signal intensity, signaling a normal force. When shear forces are applied, the three fibers will reflect different signal intensities, signaling uneven application of forces. The current design allows for maximum forces of 2.5 N (axial force) and 1.7 N (radial force) with a resolution of 0.04 N, making it suitable for suturing purposes but without enough force range for laparoscopic procedures and palpation. In connection with this, Qasaimeh et al. (2007) conceptualized a tactile optical sensor for integration with catheter tools to measure insertion forces and contact tissue compliance during endovascular surgeries. The sensor was designed based on the Fabry–Perot optical concept but with newly designed deflecting elements as simply and hybrid supported beams. The silicon-based simply supported beams were employed for measuring contacting forces as a function of light intensity modulations. The proposed hybrid beams were designed as cantilever beams supported by elastomers at their other ends, which were dedicated to measuring the compliance of contacting tissues. Although the design and the simulation work showed promise, the device is yet to be fabricated using MEMS technology and characterized experimentally.

In Ahmadi et al. (2011)’s MIS tissue manipulation and palpation sensor design, optical fiber tactile sensors were chosen for their compatibility with MRI devices. Because ferromagnetic metal components of traditional sensors interfere with MRI magnetic fields and distort MRI images, electrical wires and metals within tools constrain MRI compatibility (Puangmali et al., 2008). On the other hand, optical fiber sensors do not contain these disruptive components (Konstantinova et al., 2014). Especially with the recent MRI advances that allow MR imaging to scan and process in real-time during operations, MRI compatibility is important (Xie et al., 2013a). The sensing principle of the sensor is based on mounting three optical fibers along a deformable beam, which flexes upon applied force. Three evenly spaced protrusions along the deformable beam bend one of the three optical fibers accordingly. When a force is applied, the amount of applied force corresponds to the amount of bending and subsequent power loss of the optical fibers. The location of the force can be determined by reading which optical fiber induces the most power loss relative to the others. It was demonstrated that the sensor could locate a hard lump hidden under elastomers, simulating palpation. Static and dynamic loading was proven. However, for the 45 × 8 × 8 mm^3^ sensor to be integrated, it must be further miniaturized for integrating with MIS graspers. Furthermore, the measurement range needs to be increased in order to facilitate actual palpation procedures.

This concept was innovated early on in 1996 by Lazeroms et al. (1996), where they developed an optical-based force sensor which, based on the intensity of detected light from the optical fiber, determines the amount of stress applied to the optical fiber sensor. However, it is also noted that the bending of optical fiber in this design leads to a loss of light intensity, which can also result in a loss of accuracy and misleading measurements. Despite the successful proof of concept, miniaturization is also needed.

**FIGURE 3:**
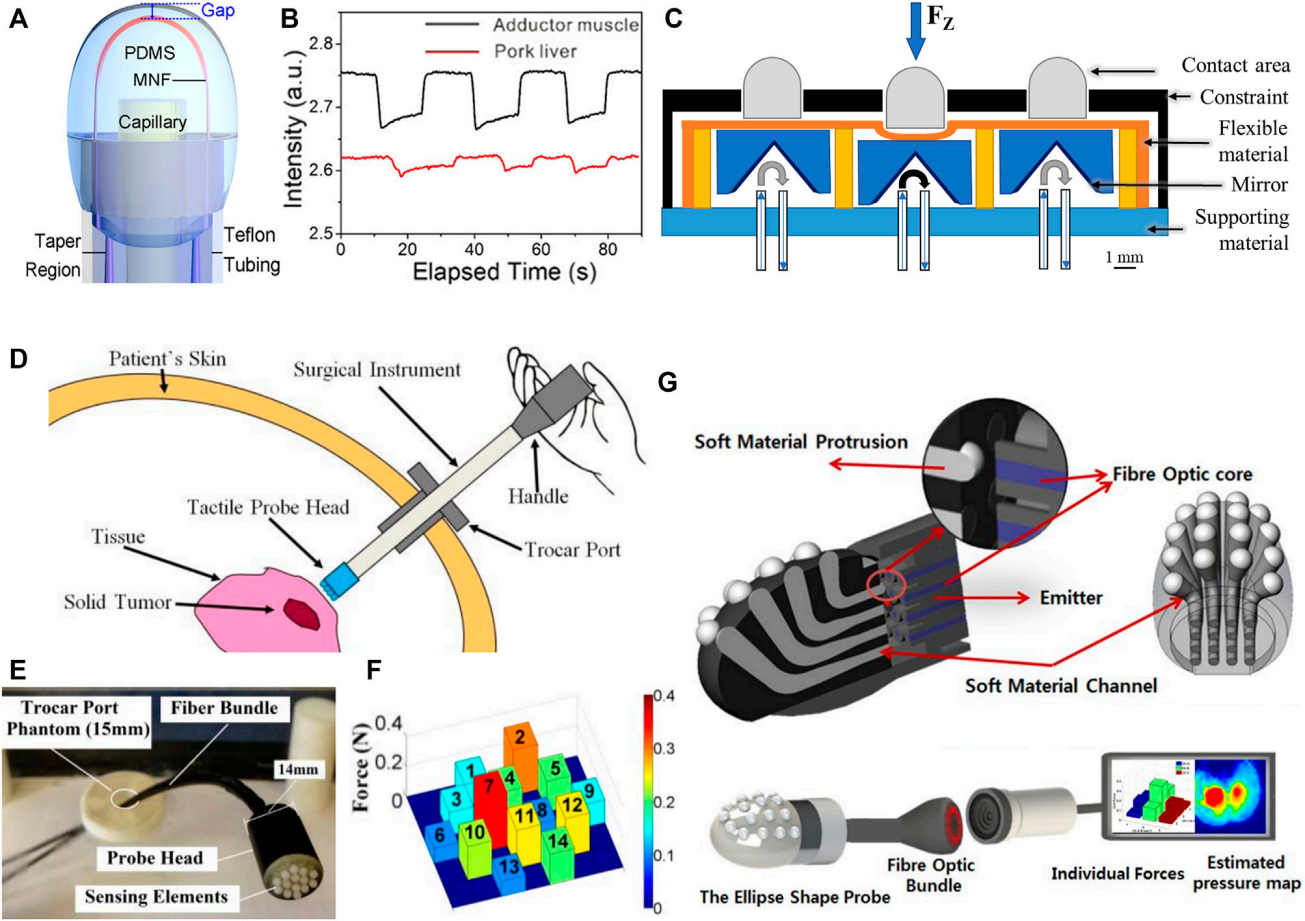
Optical-based tactile sensors for MIS. **(A)** Schematic of the micro/nanofibers (MNF)-embedded compact tactile sensor. **(B)** Response of the sensor as intensity curves corresponding to the adductor muscle of fresh mussel meat and pork liver at a pressing depth of 400 μm (Tang et al., 2021). Reprinted with permission. Copyright (2021) American Chemical Society. **(C)** Detailed sensor design of the developed fiber optics tactile array showing one fiber bundle for transmitting light and a second one for receiving light at each sensing unit (Xie et al., 2013b). **(D)** Schematic of the operation of the proposed tactile probe head. **(E)** Actual image of the probe head prototype passing through a trocar port. **(F)** Demonstration of the force feedback from the MRI-compatible tactile probe head (Xie et al., 2014). ^©^ 2014 IEEE. Reprinted, with permission. **(G)** Schematic of the ellipse shape probe utilizing optical tactile array and the camera acquisition system (Back et al., 2015). ^©^ 2015 IEEE. Reprinted, with permission.

Recently, Tang et al. (2021) presented a compact tactile sensor based on optical micro/nanofibers (Figure 3A). Based on light intensity change from the slight pressure-induced bending of the u-shaped fibers, the sensor showed the ability to discriminate objects and tissues (fresh mussel meat) based on hardness (Figure 3B). This novel sensor demonstrated pressure-sensing sensitivity as high as 0.108 mN^−1^ with a resolution of 0.031 mN.

In another study, Xie et al. (2013b) integrated a fiber optics tactile sensor array onto a tissue palpation probe for MIS instead of a grasper. Light intensity modulation was used for its versatility, easy fabrication, and inexpensive production cost. Each of the 12 independent sensing elements consisted of a ball-shaped tip contact area, with two mirrors placed at 90-degree angles to reflect light from transmitting to receiving fiber. Under this design, an applied force led to a decrease in mirror and fiber distance, therefore increasing the reflected light intensity. In the study, one photo-sensitive camera was used to detect changes in light intensity across all 3 × 4 sensing elements (covering 12 × 18 mm^2^), which was then processed through MATLAB to estimate the location and magnitude of the applied force after calibration. A detailed diagram is provided in Figure 3C. The design remains MRI-environment friendly, and tests have shown it is feasible to detect small nodules. The one-camera design lowers production costs and provides sufficient sensing resolution. Likewise, miniaturization is also a challenge that needs to be addressed for real MIS applications.

Xie et al. (2014). presented another circular tactile probe head consisting of 14 elements (Figures 3D–F) (Xie et al., 2014). A similar design of using a single camera was tested, and forces from 0 to 0.5 N were measured with a resolution of 0.05 N. Accurate measurements for frequencies up to 10 Hz were proven. However, the accuracy of sensing elements was slightly affected by the hysteresis effect of the latex rubber used as the flexible structure between the supporting material and sensing tip and the light signal loss by fiber bending and connection. This indicates that further work is needed to synthesize rubbers with faster recovery times and fibers that are less impacted by bending and connections. 8 mm elastic nodules were embedded within a lamb kidney, and a visual map was created to sense uneven forces. Concentrated forces on the map indicated the location of hidden nodules, but sometimes non-nodule locations also indicated uneven force distributions. Nevertheless, although the proof of concept was shown, miniaturization is still needed to make the 14 mm probe head fit within the 8 mm trocar port within MIS operation.

In an attempt to simplify the optical tactile sensor further, Back et al. (2015) used the Bernoulli principle to amplify the sensitivity of the sensor in a soft material light channel network. A camera is used at the end of the multi-core optic fiber network to measure changes in light intensity caused by contact forces. Such a design eliminated the need to attach reflectors to each sensing element, making it easier to fabricate and personalize based on different MIS equipment requirements. The design can be fabricated through 3D printing and casting of soft materials. The final design consisted of an ellipse-shaped probe with 16 Bernoulli-shaped-based sensing elements attached to light emitters and detectors. With information regarding the changes in light intensity, which reflected the amount of force applied onto each soft material elastomer, individual forces could be measured, and pressure maps could be visualized (Figure 3G). An average measurable force range of 0–1.622 N was determined with 97% accuracy. High-speed cameras with higher resolution are needed to reduce the noise further and to increase frame rates for optimizing the design.

As opposed to using light intensity as a function to measure applied force, fiber Bragg grating (FBG) sensors employ wavelength-encoded information to determine the force applied onto the optical tactile sensor. This concept was chosen for Song et al. (2011)’s FBG MIRS sensor because the application of FBG renders the system immune from inherent power fluctuations and connection losses. The final system included 7 degrees of motion for the MIRS arm, and the optical force sensor was located between the grasper and joint. The *X*- and *Y*-axis FBG detection satisfied the criterion of 0–10 N measuring range and 0.2 N resolution. However, the *Z*-axis detection did not satisfy the criterion. The force measured by the *Z*-axis sensor was less than expected, which required scaling to produce accurate force measurements. However, the noise was also scaled, rendering the system resolution inadequate. It was discussed that the system could still be used as a warning system if excessive force was applied. Further experiments involving visual and physical haptic warning systems are said to be further tested. In another minimally invasive application, Chmarra et al. (2006) developed TrEndo, a low-cost optical-based tracking device that measures MIS instrumental translational and rotational movements. Based on the optical measurements made by the sensors, the simulation is able to guide trainees through gimbals on the tools, redirecting them to the correct medical procedure. Motion analysis is an important assessment tool to determine whether the trainee surgeon has effectively completed a virtual surgery with accuracy and efficiency. By integrating force feedback into these simulations, trainees can become more acquainted with corrected movements on the fly (Chmarra et al., 2007).

Optical tactile sensing technologies have been commercially used for over 60 years in telecommunications and the military and, increasingly, in biomedical applications. They are well known for their versatility in being miniaturized and shaped with a little compromise on sensitivity and bandwidth (Lee, 2003). Optic fibers are compatible with sterilization, chemicals, and electromagnetic interference and are highly resistant to fluctuations in temperature. However, they are more expensive than their non-optical counterparts and are more complex in fabrication and installation. Despite this, the technology has already been implemented in endoscopes and laser surgery. Peirs et al. (2004) implemented optical fibers in MIS suturing devices that could detect uneven force application but lacked sufficient force range for LS and palpation. Meanwhile, Ahmadi et al. (2011) fabricated optical fiber sensors that could locate and detect hard lumps under elastomers but lacked miniaturization and integration. Likewise, Xie et al. (2013b) were able to show great detection potential for a 3 × 4 optical tactile array but also fell short of miniaturization for MIS applications. Other researchers have made improvements to optical tactile sensing technologies, such as component simplification, noise reduction, and force detection for several degrees of motion. For optical tactile sensing technology to become prominent in LS, researchers will need to prove that miniaturization and detection accuracy can go hand in hand. In addition, cost-saving production methods and simplification of the setup can go a long way to making this technology more MIS-applicable.

As the absence of haptic and tactile information in MIS results in sub-optimal treatment, restoring the touch sensation to surgeons *via* force sensors has become a shared research interest among many researchers. Among the different force sensing modalities evaluated for MIS, electrical-based sensing is the most attempted due to the ease of fabrication and a smaller number of components. Additionally, electrical sensors can be miniaturized using silicon fabrication techniques, allowing small sensing elements to be constructed and also combined with the required electronics as the MEMS industry supports. Still, limitations such as temperature dependence and hysteresis hinder their adoption with MIS. On the other hand, optical-based sensing shows good sensitivity and response time to static and dynamic loadings. Moreover, being electrically passive makes it compatible with MRI. However, optical systems require careful installation and calibration procedures. Therefore, their implementation in MIS remains limited. Overall, these conventional sensing techniques are robust and can function over a wide range of pressures and temperatures. Additionally, sensitivities and working ranges can be precisely designated based on the selection of material and fabrication techniques. Table 2 presents a comparison between the conventional tactile sensing technologies for MIS.

**TABLE 2:** Comparison between the conventional tactile sensing technologies for MIS.

	Piezoresistive sensors	Piezoelectric sensors	Capacitive sensors	Optical sensors
Hysteresis	High	Low	High	Low
Temperature dependence	Yes	Yes	No	No
Humidity dependence	No	No	Yes	No
Power consumption	High	Very low	Low	High
Linearity	Good	Good	Fair	Good
Cost	Very low	Low	Medium	High
Electronics	Simple	Simple	Intermediate	Complex
Static pressure capability	Yes	No	Yes	Yes
Advantages	- Small size	- Small size	- Better stability and higher sensitivity than the two other electrical sensors	- High spatial resolution
	- Easy multi-axial force measurement	- No moving parts	—	- Compatible with MRI scanners
	- Simple readout circuits	- Self-powered	—	—
	- Low noise	- High bandwidth	—	—
Limitations	- Sensitive to EM noise	- Suitable for the measurement of dynamic loads only	- Sensitive to EM noise	- Sensitive changes in light intensity due to cables bending
	- Trade-off between the sensitivity and the stiffness of the structure	- Requires a charge amplifier	- Signal processing complexity	- Requires precise alignment and packaging of fibers to maintain the calibration
	- Trade-off between scaling down and power consumption	—	- Requires careful circuit design to reduce the effects of parasitic capacitance	—

## 5 Emerging Tactile Sensing Technologies

As shown earlier, the conventional tactile sensing technologies, i.e., silicon MEMS devices based on electrical and optical sensing principles, have been utilized in several MIS tools for restoring the tactile sensation. In parallel, some new tactile sensing techniques are showing great potential in various fields of engineering. An emerging type of force sensor was developed by embedding liquid metals within elastic materials. Under mechanical deformation, force detection results mainly from the flow of liquid substances through the microchannels resulting in a change in the response of the sensor. Another novel sensing technique utilizes an imaging system to track the induced deformation of the sensing diaphragm, or skin, by utilization of a camera system with a high resolution and a fast response time. Both techniques have been investigated for robotics and biomedical fields, in which they showed great potential and increased sensitivity over the conventional tactile sensing methods. Utilizing the advancements of such emerging techniques can bring up further enhancements to MIS tactile sensing applications. The following is an MIS-oriented discussion of the emerging tactile sensing technologies, i.e., microfluidic and imaging tactile sensing.

### 5.1 Microfluidic Tactile Sensing

Advances in flexible electronics have pioneered new classes of soft, elastic, skin-like sensors with a substantial potential over conventional, rigid devices for application in wearable electronics (Tang, 2007), health monitoring (Takei et al., 2015), soft robotics (Lu and Kim, 2014), and artificial e-skins (Kanao et al., 2015). Aside from all-solid-state sensors, the novel approach of “liquid-state electronics” based on encapsulating fluids within thin elastomeric structures facilitates highly flexible and stretchable sensing devices (Ota et al., 2014). For instance, a vibration sensor enclosing sodium chloride (NaCl)-filled chambers interprets the mechanical, vibration-induced motion of ions in the electrolyte for detecting vibrations over a wide frequency range (Kim and Seo, 2008). Curvature sensors consisting of soft elastomers and conductive liquids were realized for softer-than-skin electronics (Majidi et al., 2011). Another liquid-based sensor, a reversibly stretchable wireless strain sensor comprising an elastic liquid metal patch antenna heterogeneously integrated to a simplified radiofrequency (RF) transmitter provides remote sensing of high strains up to 15% over large surfaces close to 100 cm^2^ in size and motion detection of huge movable parts (Cheng and Wu, 2011). Besides, stretchable electrodes, such as liquid metals (LMs) (Hu et al., 2007; Kim et al., 2008; Hotta et al., 2012) and conductive carbon grease (Maleki et al., 2011), have been used as soft electrodes and electrical interconnects for applications requiring flexible electrical components.

For tactile sensing, Wettels et al. (2007) developed a biomimetic tactile sensor array, called the BioTac (SynTouch, Los Angeles, CA), aiming towards enhancing the performance of robotic and prosthetic hands. Consisting of a solid central core, a layer of sensing electrodes, a weakly conductive fluid, and an outer silicone elastomeric skin, the proposed sensor can indicate the direction and magnitude of the force and the contact point and shape of the object from the resulting impedance pattern during grasping tasks (Wettels et al., 2008). Similarly, dynamic capacitive pressure mapping was realized using a continuous thin fluidic layer embedded in a compact transparent flexible 200 μm thick package (Li et al., 2014; Nie et al., 2015). Furthermore, galinstan-PDMS composite arrays formed robust and deformable pressure-conductive rubber-based sensors (Oh et al., 2019).

Besides improving the flexibility of the tactile sensor, the deformation or movement of liquids within the sensing elements can be anticipated as a tactile sensing transducer. On one side, the sensing elements of droplet-based tactile sensors only involve microdroplets of liquid, including DI water (Gutierrez and Meng, 2011), mercury (Bakhoum and Cheng, 2010), ionic liquids (Nie et al., 2014a), and dielectric oil (Takahashi et al., 2012). In one implementation, a 25/75% electrolyte/glycerol droplet was sandwiched between two flexible polymer membranes with a conductive coating (Nie et al., 2012). With a highly capacitive electric double layer, the sensor had an ultrahigh-pressure sensitivity of 1.58 μF kPa^−1^ and a resolution of 1.8 Pa. More importantly, the sensor was fabricated by low-cost one-step laser micromachining. On the other side, the recent introduction of microfluidics to the field of mechanical sensing has generated alternative sensing mechanisms, flexible sensing designs, and soft matter constructs, offering a wide range of new possibilities (Pan and Wang, 2011). Indeed, microfluidics possesses several practical features, such as miniaturized sizes, easy fabrication, cost efficiency, and scalable manufacturability (Nie et al., 2014b). This section will investigate the microfluidic-based tactile sensing approach and particularly highlight its potential for MIS applications.

#### 5.1.1 Structure and Material Requirements

**FIGURE 4:**
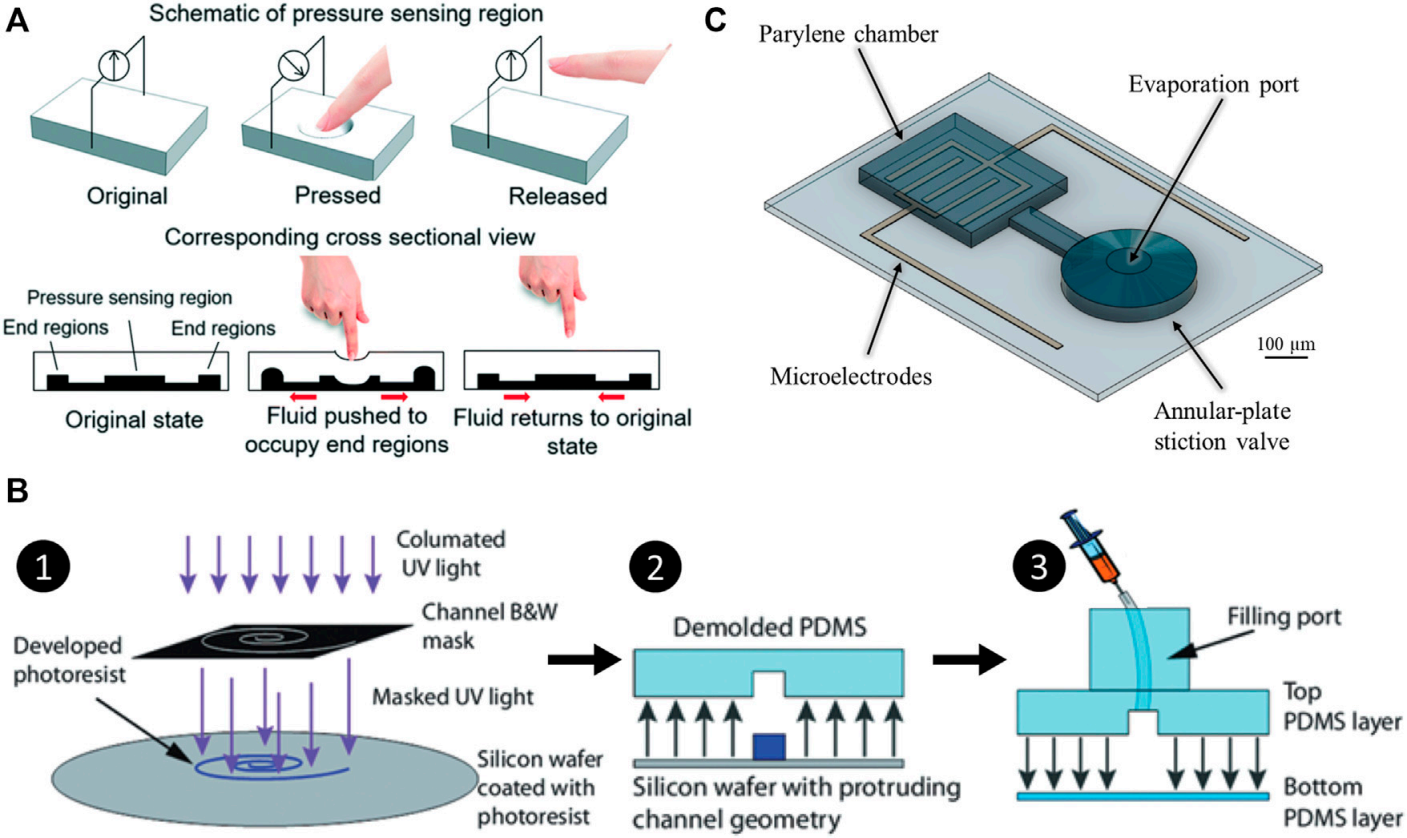
Microfluidic tactile sensing. **(A)** Working principle of microfluidic force sensing where the applied force induces a deformation of the elastomer and causes the liquid to flow inside the microchannels (Yeo et al., 2016b). Published by The Royal Society of Chemistry (RSC). **(B)** Manufacturing steps for microfluidic tactile sensors: First, a photolithography process is used to pattern extrusions on a silicon wafer coated with a photoresist, and treatment with the developer solution eliminates the non-exposed photoresists. Next, the elastomer is poured over the patterned silicon wafer, resolving microchannels on the soft layer when demolded. Lastly, an additional bottom layer is added to seal the channel, and a working liquid is injected into the microchannels through a filling port (Codd et al., 2014). Reprinted with permission. Copyright Patrick J. Codd. **(C)** Concept of the impedance-based microfluidic tactile sensor with an electrolyte-filled microchamber forming the sensing unit and an in-line stiction valve for liquid encapsulation (Gutierrez and Meng, 2010).

Microfluidic force sensing elements can be formed by injecting a minute amount of liquid medium into elastomeric microchannels, at which external loadings induce fluid displacement/deformation of working liquid (Figure 4A). The applied force can then be determined by characterizing the corresponding alteration in the electrical or optical properties of the liquid sensing medium. Working fluids with low viscosity facilitate both rapid mechanical responses to external stimuli and low hysteresis for sufficient transient and dynamic tactile sensing (Tonti, 2013). Additionally, low vapor pressure is highly desirable to ensure physicochemical stability in conductivity and viscosity over wide electrical potentials, operating temperatures, and humidity levels. Other material considerations are directly related to the detection principles employed. For instance, resistive sensing would require highly resistive liquid to reach higher sensitivity, whereas capacitive sensing considers the high permittivity of the liquid as one primary selection criterion. Table 3 provides a summary of the properties of major commercially available sensing liquids.

**TABLE 3:** Summary of properties of major tactile sensing working liquids.

	Melting point (^o^C)	Dynamic viscosity (Pas)	Electrical conductivity (S∙m^−1^)	Vapor pressure (Pa)	Toxicity level
Mercury (Surmann and Zeyat, 2005; Silverman et al., 2006)	−38.8	1.55 × 10^−3^	1.04 × 10^6^	16.3 × 10^−6^ (at 20°C)	High
Gallium (Morley et al., 2008; Khoshmanesh et al., 2017)	29.8	1.96 × 10^−3^	6.73 × 10^6^	∼10^−35^ (at 29.9°C)	Low
Galinstan (Surmann and Zeyat, 2005; Khoshmanesh et al., 2017)	13.2*	2.40 × 10^−3^	3.46 × 10^6^	<100 × 10^−12^ (at 25°C)	Low
EGaIn (Eutectic Gallium–Indium) (Zrnic and Swatik, 1969; Silverman et al., 2006)	15.5	1.69 × 10^−3^	3.40 × 10^6^	—	Low
1-Ethyl-3-methylimidazolium dicyanamide (MacFarlane et al., 2002; Cheng et al., 2013)	−21	21 × 10^−3^ (at 20°C)	5	—	Intermediate
1-Ethyl-3-methylimidazolium tricyanomethanide (Nie et al., 2014a)	−11	18 × 10^−3^ (at 25°C)	1.8	—	High
1-Ethyl-3-methylimidazolium ethyl sulfate (Noda et al., 2010)	—	97.58 × 10^−3^	0.398	0	High
DI water (Khoshmanesh et al., 2017)	0	1 × 10^−3^ (at 25°C)	<5 × 10^−4^	3.169 × 10^3^ (at 25°C)	Nontoxic (biocompatible)
Ethylene Glycol (Li et al., 2014)	—	16 × 10^−3^ (at 25°C)	1.07 × 10^−4^	7.5 (at 20°C)	High
Propylene Glycol (Li et al., 2014)	—	40 × 10^−3^ (at 25°C)	1 × 10^−5^	17 (at 20°C)	Low
Glycerol (Li et al., 2014)	—	0.934 (at 25°C)	4.25 × 10^−6^	<0.33 (at °C)	Low
Ethanol (Li et al., 2014)	< −100	1.203 (at 25°C)	—	5.83 × 10^3^ (at 20°C)	Low

* Galinstan exhibits significant supercooling behavior with a freezing temperature of −19°C.

Among many liquids, Gallium-based LMs maintain a liquid state at room temperature and serve as non-toxic alternatives to mercury (Liu et al., 2012). Due to high surface tension, high electrical conductivity, low toxicity, and low viscosity, eutectic alloys of galinstan (68.5% Ga, 21.5% In, and 10% Sn) and Eutectic Gallium–Indium (EGaIn: 75.5% Ga and 24.5% In) have been the two most popular LM alloys used for microfluidic force sensors or as substitutes for wires (Dickey et al., 2008). LM-based force sensors are highly appreciated for being intrinsically immune to cracks and fatigue, making them suitable for conformal wrapping and large repetitive strains. In addition to LMs, room temperature ionic liquids (ILs) have been used to form highly deformable pressure sensors (Zuo et al., 2010). Generally, ILs manifest a negligible vapor pressure, high boiling point, nonflammability, and excellent chemical stability in contact with both water and air (Wong et al., 2008). Due to their higher resistivity, ILs are ideal for maximizing the variation in absolute resistance and minimizing the influence of random variations at the solid/liquid interface. Graphene oxide (GO) nanosuspension is another conductive fluid with low surface tension that occupies the specific shape of microchannels in contrast to high surface tension liquids.

The microfluidic-based circuits can be easily designed and fabricated using micro-channel infusion or liquid metal printing. Printing technologies, being more convenient, have relatively limited fabrication precision. Meanwhile, microfluidic technologies enable a stable fabrication of uniform and sealed LM-based circuits (Figure 4B). Regarding elastomers, PDMS has been the most explored structural material in microfluidics due to its high elasticity and biocompatibility, along with the ability to define high-precision microchannels with micrometer resolution (Duffy et al., 1998). PDMS offers the advantages of non-toxicity, chemically inert nature, robustness, high degrees of flexibility, low cost, simple processing techniques, and impermeability to liquids (Quake and Scherer, 2000). Thus, PDMS protects the embedded sensor electronics from environmental factors. Besides, silicone rubber (Ecoflex: polybutylene adipate terephthalate biodegradable copolymer), polyimide (PI), and polyethylene terephthalate (PET) elastomers were reported as well for their superior flexibility and conformability. Depending on the mixing ratio of the base material and the curing agent, the simple fabrication method of molding offers precise control over the elastomeric mechanical properties, e.g., Young’s modulus and Poisson’s ratio. Like electrical-based sensors, resistive- and capacitive-based tactile sensing approaches are both applicable to microfluidics, while triboelectric mechanism serves as an alternative to the self-powered mechanism of piezoelectricity.

#### 5.1.2 Impedance-Based Microfluidic Tactile Sensors

When using ILs in microfluidic circuits, electric double-layer capacitors form at the electrode/electrolyte interfaces. This capacitive interaction generates ionic current flow by non-Faradaic mechanisms (Robblee and Rose, 1990). The circuit model of the interface can be represented by a Helmholtz double-layer capacitance, C_dl_, in parallel with resistance to charge transfer, R_ct_ (Randles, 1947). Therefore, alternating current (AC) becomes very useful towards preventing ions accumulation at the electrodes. In this tactile transduction mechanism, the change in electrolyte volume impedance around the electrodes corresponds to the mechanically induced deformation by the applied loading.

The first demonstration on microfluidic-based tactile sensing was achieved in 2009 by Tseng et al. (2009). The proposed microfluidic tactile sensing transducer consisted of a top PDMS layer with a hemispherical reservoir filled with NaCl and a bottom layer of PI containing a microchannel laying on a sensing electrode pair. By pressing on the reservoir, the electrolyte would be forced to flow into the microchannel. Accordingly, the output signal gets triggered continuously until the stimulus is removed, mimicking the function of slow-adapting receptors in human skins. The device showed a linear response, a sensitivity of 6.06 mV N^−1^, and an operating range of 0–1.8 N. Towards developing multimodal biomimetic skins, the sensor fabrication process can be made suitable for common artificial skin materials such as silicone rubber.

Later, Gutierrez and Meng (2010) employed electrochemical-MEMS technologies to fabricate a perylene-based transducer filled with Deionized (DI) water as an electrolyte. High-sensitivity measurements of interfacial contact forces were enabled by a transducer square chamber, whereas a circular chamber served as a valve for liquid injection and self-sealing (Figure 4C). The potential of such a physical transducer was demonstrated through biomimetic mechanotransduction along interconnected channels and out-of-plane microelectrode actuation through the electrolysis of water. In moving forward, a more optimized sensor design with fluidic access ports and thin-film platinum electrodes was reported (Gutierrez and Meng, 2011).

For pressure mapping, Chossat et al. (2015) developed a novel manufacturing method of soft skin sensors having a netlike microfluidic structure. The skin sensor incorporated Ecoflex substrate with multiple embedded microchannels and twelve casted electrodes. To completely fill the microfluidic channels, 1 ml of IL (1-ethyl-3-methylimidazolium ethyl sulfate) was injected through a silicone mesh layer into the first layer. With holes 170 times smaller than the microchannel’s cross-sectional area, the silicone mesh layer allowed the air to vent out while being small enough to enclose the IL inside and prevent its leakage by surface tension. Following the injection of the working liquid, the rest of the layers were stacked on top. The microfluidic sensing element was modeled as a matrix of resistors such that each channel section represents a resistor and each connection between the channels represents a node. This matrix-type sensor utilized electrical impedance tomography to detect surface contacts without adding internal wiring. Data acquirement was based on measuring electrical potentials between all the electrodes except two electrodes to which an alternating current is supplied, then changing the two AC electrodes until all electrode combinations are involved. The simple numerical method of weighted filtered back-projection was employed to validate the skin sensor operational concept. Eventually, magnitudes and locations for both single and multiple loading conditions were graphically displayed.

Careful design of impedance-based microfluidic sensors enables high-sensitivity force measurements with fast response time and decent spatial resolution. An AC voltage supply powers this type of sensor; otherwise, the ions of the IL will start accumulating in the region near the sensing electrodes. While force-induced impedance changes are highly predictable and reproducible, microfluidic tactile sensors based on resistance change are more commonly addressed due to the well-established foundation in solid-state sensors and less complicated circuits designed for the direct current (DC) supply.

#### 5.1.3 Resistive-Based Microfluidic Tactile Sensors

In microfluidic resistive tactile sensors, the elastomeric deformation contributes to changes in the cross-sectional areas and length of the microchannels, resulting in a measurable resistance change along the electrolyte-filled microchannels. Indeed, the degree of microchannel deformation under a given load and, by extension, the overall sensor sensitivity is governed by the microchannel geometry, elastomer material properties, and depth of the embedded channels within the elastomer (Hammond et al., 2014a).

Park et al. (2010) investigated the influence of microchannels design on the performance of stretchable, hyperelastic, softer-than-skin pressure transducers. Three structures of EGaIn-filled elastomeric microchannels were evaluated: a spiral-shaped channel for pressure sensing only, a serpentine-shaped channel with reservoirs for increased sensitivity, and a strain gauge for simultaneous sensing of stress and directional strains. All prototypes were fabricated by casting Ecoflex in molds produced with either a 3D printer (250 μm—2 mm channel dimensions) or a maskless fabrication method that combines direct laser writing with soft lithography (25–300 μm). Theoretical and experimental studies validated that the pressure working range can be controlled by varying the aspect ratio (height/width) of the microchannel cross-section. Additionally, the change in the electrical resistance was less significant in channels deeper or farther away from the pressure center. With this in mind, microchannels embedded deep within the elastomer only measured stretch events and not pressure. Alternatively, spiral-shaped microchannels embedded close to the elastomer surface could only detect pressure since, under a uniaxial stretching, the electrical resistance increase in one direction was canceled out by a reduced resistance in the perpendicular direction. Based on those findings, the authors presented an artificial skin sensor incorporating two orthogonal strain-sensitive layers and a circular patterned pressure-sensitive layer (Park et al., 2011). The proposed multilayered EGaIn-filled Ecoflex-based sensor can distinguish the three different stimuli of *X*- and *Y*-axis strains and *Z*-axis pressure. Characterization tests of a 25 × 25 × 3.5 mm^3^ working prototype comprising microchannels (with 200 × 300 µm^2^ cross-section) showed strain sensing linearity even beyond 100% strains but nonlinearity in pressure sensing. Nevertheless, the sensor could sense a minimum pressure of 15 kPa approximately (Park et al., 2012).

Microfluidic hyperelastic skins capable of strain and pressure sensing showed significant potential for lightweight, flexible electronics, i.e., wearable devices. They have been demonstrated for measuring angels and contact forces of joints, such as fingers (Kramer et al., 2011a), ankles (Park et al., 2014), and robotic joints (Noda et al., 2010). Other researchers introduced sensor-embedded gloves for detecting human hand motions and tactile pressures (Hammond et al., 2014b). A wireless smart insole integrated with a stretchable microfluidic sensor was developed for gait monitoring (Low et al., 2020). Motion sensing suits with integrated microfluidic skins fulfilled the goal of monitoring lower limb (Mengüç et al., 2013) and gait (Mengüç et al., 2014) biomechanics. Polipo pressure-sensing system, consisting of multiple pressure microfluidic sensors with spiral-shaped channels of galinstan, was developed to monitor the interaction between the person and the spacesuit during extra-vehicular activity, detecting pressures as low as 5 kPa (Anderson et al., 2015). Besides, utilizing miniaturized sensors and linking individual sensors with flexible materials allows for accurate placement and proper pressure distribution characterization of the body (Shen et al., 2018). Measuring arterial parameters, e.g., post-exercise response, using a 5 × 1 microfluidic resistive transducer array offered a low-cost and simple arterial health assessment (Hao et al., 2020). Similarly, a thin, transparent wearable tactile keypad was developed using EGaIn-filled microchannels embedded in a PDMS film (Kramer et al., 2011b). With this stretchable artificial skin, the user could write any combination of alphabetic letters by pressing on channel intersections; each triggers one of the twelve keys. Recently, a prosthetic hand equipped with a sensorized fingertip gained the capability of surface feature recognition and grasped object slip prevention (Abd et al., 2020).

With the goal of providing real-time pressure feedback during neuroendoscopy, Codd et al. (2014) mounted a flexible pressure-sensing polymer skin on an endoscope operating sheath. The developed sensing skin incorporated a 3 × 3 array of identical EGaIn-filled spiral PDMS microchannels as pressure transducers. In *ex vivo* tests, the sensorized endoscopic tool was manually pressed against the cortical surface of an adult sheep brain and then was introduced perpendicularly into the organ to simulate a transcortical endoscopic trajectory. The authors displayed the amount of force applied during operation using a programmed color-coded graphical user interface. Hence, the smart surgical tool could avoid impending collateral damage, particularly during minimally invasive brain and spine surgery. Subsequently, the sensor’s biocompatibility and sensitivity were further enhanced by replacing the metal liquid with NaCl-saturated glycerol (C_3_H_8_O_3_) and changing the microchannels pattern into a serpentine structure on top of a stress intensifying layer, respectively (Arabagi et al., 2016). In another work, this sensing concept was reduced to point-pressure measurements, which facilitated contact force and angle sensing at the tip of endoscopic instruments and standard microsurgical dissection tools (Arabagi et al., 2013). Experimental results showed that the soft sensor could accurately detect contact angle and contact force within ±2° and ±6 g on average, respectively.

Microfluidic resistive tactile sensors have also enabled the detection of distributed static and dynamic loads. In one study, Gu et al. (2013a) presented a PDMS-based sensor comprising a rectangular microstructure on top of five evenly distributed electrolyte-enabled transducers. After careful selection of proper electrolytes and the AC signal operation frequency, both the electrolyte–electrode interface impedance and the electrolyte capacitance were neglected. A prototype filled with 1-ethyl-3-methylimidazolium dicyanamide underwent further performance evaluation and testing against PDMS samples having voids inside, demonstrating an efficient acquisition of spatially varying elasticity/viscoelasticity of heterogeneous soft materials (Cheng et al., 2013). Through dynamic characterization, the amplitude ratio and the phase shift between the sinusoidal load and deflection of the device at different frequencies were analyzed into dynamic stiffness and damping of the device (Gu et al., 2015). Then, the system-level parameters of the second-order mechanical system device were extracted. Also, the authors presented a novel experimental technique of concurrent spatial mapping for spatially varying elasticity measurement of heterogeneous soft materials using a single microfluidic-based sensor (Gu et al., 2013b). In their demonstration, a rigid probe with controlled displacements was used to press a testing specimen against the device that decodes the distributed continuous loading into the specimen’s spatially varying elasticity. The proposed sensor can potentially deliver haptic feedback during MIS tissue manipulation and palpation, together with many design benefits, including fabrication simplicity, ease of electrolyte injection, and small footprint.

Shear forces are particularly important for locomotion (i.e., traction) and manipulation (i.e., sensing grasp failure). Towards microfluidic shear force sensing, Vogt et al. (2012) developed a soft multi-axis force sensing skin. The design consisted of a rigid plastic force-post atop of Ecoflex microchannels embedded with EGaIn to increase the force-induced deformation of the channels. With three sensing elements arranged in a star pattern, the sensor could decipher in-plane and a normal force with *X*-, *Y*-, and *Z*-axis sensitivities of 37.0, -28.6, and 27.8 mV/N, respectively. The parametric modification of the microchannels’ width, force-post diameter, and height was also studied (Vogt et al., 2013). The working prototype showed a nonlinear response and high hysteresis level in pressure sensing, although having linear and repeatable strain responses up to 180%. The nonlinearity was associated with the nonlinear areal reduction rate of the rectangular microchannels under loading. Thus, a simple yet effective solution to improve sensing signals proposed changing the physical geometry of embedded microchannels in liquid embedded hyper-elastic pressure sensors (Park et al., 2012). Both simulations and experiments illustrated the significant influence of the channel’s cross-sectional geometry on the pressure-sensing linearity, sensitivity, and hysteresis. Channels with a concave triangular cross-section exhibited the best performance among four different channels. Ultimately, microfluidic shear force sensors are promising for wearable devices, where loads subjected to human skin are critical for comfort.

A PDMS-based microfluidic device capable of detecting distributed shear loads was introduced by Yang et al. (2014). The 2 × 3 sensing array prototype involved a shear-loading bump that translates shear loads into normal loads of two opposite directions, hence inducing opposite geometrical changes to the two microchannels underneath. Furthermore, torques could also be captured from unique torque-induced resistance changes in the two side transducers (Yang and Hao, 2015). Conventional molding and soft lithography techniques were combined to fabricate the prototypes. Later, a 3 × 3 transducer array of the same sensing structure was used to investigate the impact of using a sensor-assisted robotic arm on tissue palpation (Yang et al., 2016a). The system considered each sensing plate and the portion of tissue underneath as two springs in series. Then, the stiffness ratio between the tissue and the sensor was predicted by establishing a relation between the sensor’s deflection and the indentation depth, defined as deflection slope. The sensor was proven suitable for tumor localization of well-prepared tumor tissue phantoms (Yang et al., 2015) and mice tumor tissues (Yang et al., 2016b). Although the final microfluidic-based tactile sensor prototype was more immune to misalignment errors, an accurate tumor identification requires a threshold value of the slope difference in a region to be assigned beforehand.

In the same context, Shi et al. (2012) developed a piezoresistive normal and shear force sensor containing liquid metal as gauge material, which simplifies the sensor reading for pure resistance under DC voltage in contrast to using ILs. Within a 2 mm thick PDMS structure, the sensor encompasses 100 µm wide gauges formed out of Coollaboratory Liquid Pro (a liquid metal alloy of gallium, indium, rhodium, silver, zinc, and stannous). Since pressing a microfluidic strain sensor reduces its cross-sectional area, force sensing can be realized in vertical and lateral directions based on the strain sensor tilting angle. The sensor could differentiate between shear and normal forces by combining a symmetric pair of oppositely tilted gauges (30°) without requiring a bump. A similar structure was utilized as the sensing element of an artificial hair cell sensor (Shi and Cheng, 2013). Conversely, a study on hysteresis was carried out with screen-printed EGaIn as a gauge material this time (Shi et al., 2016a). Testing the proposed sensor at different loading speeds showed the significant influence of the force loading rate on the performance of the device. This cumulative work highlighted that microfluidic-based force sensing offers improved flexibility and durability without compensation for the sensitivity but at the cost of a higher hysteresis effect.

In Yin et al. (2017)’s work, a bioinspired, thin, flexible shear force sensing skin for tactile sensing applications was fabricated based on EGaIn-filled PDMS strain gauges. The sensor skin was wrapped around a rigid artificial fingertip. Under shear force, one side of the skin experienced tension, while the other side got compressed and buckled, similar to a human fingertip. Furthermore, the sensor demonstrated capabilities of sensing dynamic shear force, vibration, and slippage (Yin et al., 2018). Static response experiments showed that the sensorized skin is functional over an extended dynamic range, insensitive to the applied normal force, intrinsically flexible, and immune to fatigue when subjected to repeated large strains. This shear sensing skin design stands promising for probing friction coefficient. Once appropriately calibrated, it can also be made compatible with various artificial fingertip geometries, addressing finger surface geometry variations. Ultimately, the sudden drop in the shear force’s magnitude can be used as feedback for robotic grasp regulation.

Hammond et al. (2014a) aimed to enable force feedback in micromanipulators, i.e., forceps. The developed soft, thin tactile sensor array consisted of two EGaIn-filled PDMS microstructured layers arranged in an orthogonal configuration. A two-dimensional matrix of 8 tactile pixels, also called taxels, was configured as a 2 × 4 sensing array. Prior to this work, the mechanical channel pinching phenomenon and significant sensitivity mismatches between sensing layers resulted from the microchannel geometries, which limited the sensor functionality under higher loads (Hammond et al., 2012). Therefore, the geometry and placement of conductive liquid microchannels were numerically optimized, and the sensor’s nonlinear elastic mechanics were simulated using finite element analysis. Consequently, tactile sensing experiments demonstrated an increased sensitivity to normal contact forces down to 50 mN and an improved contact localization resolution on the order of 500 μm. The motions and abstract geometries of objects imparting a force on the sensor surface were inferred by analyzing the microchannel deformation patterns.

**FIGURE 5:**
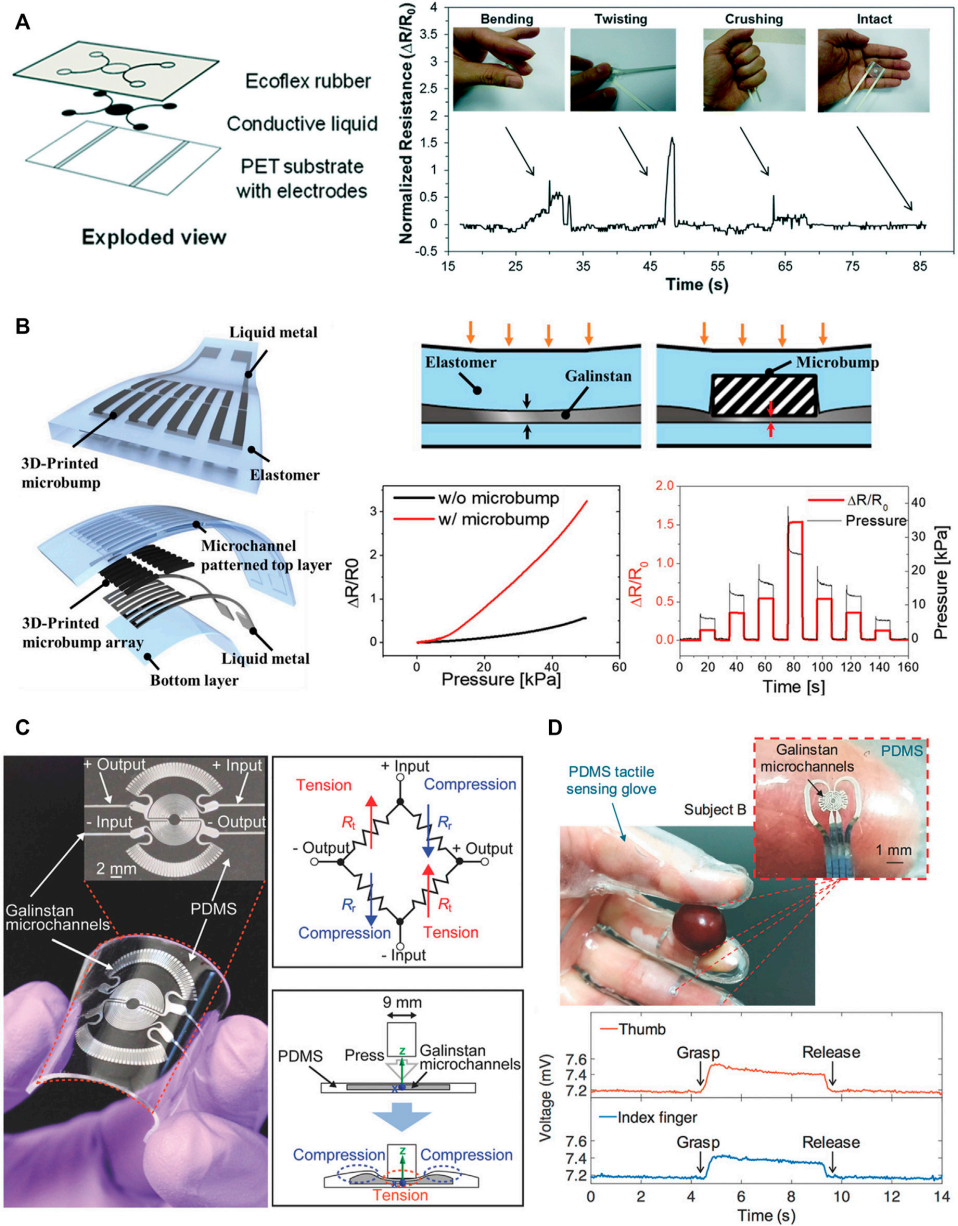
Microfluidic tactile sensing based on electrical resistance change. **(A)** Exploded view of the flexible microfluidic pressure sensor and normalized electrical resistance profile of the pressure sensor subjected to bending, twisting, and crushing during characterization (Yeo et al., 2016b). Published by The Royal Society of Chemistry (RSC). **(B)** Schematic of the 3D‐printed rigid microbump‐integrated liquid metal‐based pressure sensor showing the effect of the microbump on pressure sensitivity and the sensing response to the application of varying pressure levels (Kim et al., 2019). Copyright (2019) WILEY-VCH. **(C)** Optical image and schematics of a microfluidic tactile diaphragm pressure sensor with liquid metal Wheatstone bridge circuit. **(D)** Real-time response recorded from the corresponding thumb and index finger sensors while grasping a grape (Gao et al., 2017). Copyright (2017) WILEY-VCH.

Yeo et al. (2016a) developed a triple-state LM-based microfluidic tactile sensor by constructing an Ecoflex-PET film microfluidic assembly filled with EGaIn interfacing two screen-printed silver electrodes. This flexible sensor could distinguish different bending and compressive mechanical loads from the change in the electrical resistance. The S-shaped microchannel design with a central circular reservoir at the impact area exhibited high flexibility and durability for pressures up to 400 kPa. In a follow-up study, the microchannels were redesigned into a parallel arc structure analogous to parallel electrical circuitry, thereby reducing the overall electrical resistance and achieving a sensitivity of 0.05 kPa^−1^ (Figure 5A). Furthermore, the robustness of this microfluidic pressure sensor was demonstrated by 2,500 repetitive loading cycles and temperature variation testing between 15 and 45°C without compromising its reliability (Yeo et al., 2016b).

Subsequently, as forces tangential to the surface boast high importance in tactile sensing, the design needed additional improvements to possess such functionality. Hence, introducing a central dome at the “S-shaped” structure realizes a distinguishable response to normal forces and one-directional lateral forces, as the deformation is unique for each (Yeo et al., 2017). The sensor’s performance was experimentally validated through scanning triangular gratings at different scanning rates, different grating heights, and different protrusion diameters. The final product was flexible, robust, and easily worn by the user while allowing wireless data transfer through a Bluetooth module. Furthermore, this sensor has a promising future in Braille reading application after successfully identifying letters across a 3D printed Braille element.

While most microfluidic sensors consist of two elastomeric layers, Kenry et al. (2016) showed that Ecoflex-PDMS assembly has significantly higher peel strength than PDMS–PDMS and Ecoflex–Ecoflex candidates. The working prototype consisted of a straight microchannel filled with graphene oxide nanosuspension. Throughout the demonstration, the sensor differentiated several hand gestures and hand muscle-induced motions, highlighting the significant role of microfluidics in developing wearable diagnostic devices and real-time health monitoring.

By studying the geometry-impact of the microchannels, Shin et al. (2016) confirmed that adding solid microspheres into microchannels will significantly influence the electromechanical response to applied surface pressure in terms of improved linearity, sensitivity, and dynamic range of the microfluidic resistive sensors. Using microstructures to transfer stress is one effective way of improving the performance of microfluidic tactile sensors. By locally concentrating the microchannel deformation, Kim et al. (2019) fabricated a rigid micro-bump array using simple, cost-effective 3D printing for enhancing the pressure sensitivity of 0.158 kPa^−1^ at 50 kPa (Figure 5B). This improved performance was achieved while preserving a stable signal response, high signal recovery characteristics, and no hysteresis under cyclic loading.

Optimizing the microfluidic circuit can also improve sensing accuracy and reduce response time. For example, Goa et al. (2017) developed a wearable microfluidic diaphragm pressure sensor based on an equivalent Wheatstone bridge circuit of galinstan microchannels (Figure 5C). The micropatterned sensor comprises four primary sensing grids connected end to end: two tangential sensing grids at the center and two radial sensing grids around the periphery. By taking advantage of tangential and radial strain fields, the sensor facilitated 0.0835 kPa^−1^ sensitivity, 90 ms response time, and 0.098–800 kPa working range. Figure 5D shows a real-time response recorded from the corresponding thumb and index finger sensors while grasping-releasing a grape.

Lie et al. (2018) attempted to achieve high-pressure sensitivity by vertically arranging a 3D helical EGaIn layout inside a 24 × 12 × 5 mm^3^ hydrogel matrix. The biocompatibility and human tissue‐like mechanical property are among the advantages of hydrogels that encouraged the authors to employ polyacrylamide (PAA)‐alginate as the base material for the proposed microfluidic sensor. Additionally, hydrogel electronics dramatically fall in size and functionality when dehydrated. Recovery of the mechanical and electrical functionality can be accomplished through rehydration by simply casting into water. However, the sensor showed poor performance in distinguishing varying levels of pressure due to the high elasticity modulus of the hydrogel.

Multifunctional tactile sensors are highly desirable for wearable and robotic applications towards minimizing the number of integrated electronics. In this context, Wang et al. (2020) demonstrated both force and temperature sensing capabilities using two galinstan-based microchannels acting as sensing electrodes. While this design failed to measure the simultaneous change in pressure and temperature, two additional channels were placed away from the force sensing area, thereby decoupling temperature and force measurements using a Wheatstone bridge circuit (Wang et al., 2021). In other words, the central channels would deform significantly under mechanical loading, whereas the distanced channels only measured the temperature change. Long-term continuous cyclic loading and heating/cooling tests demonstrated the durability and repeatability of this wearable microfluidic sensor.

The fabrication of microfluidic resistive tactile sensors is not limited to the classical process of elastomer molding and liquid injection. Recently, laser-induced selective adhesion transfer was introduced as a novel, efficient method to pattern LM microchannels as narrow as 50 μm as shown in Wu et al. (2020). A femtosecond laser selectively increased the PDMS surface roughness through direct laser writing and reduced its wettability and adhesion to LMs. Then, subjecting the PDMS substrate to a bath of LM resolved micropatterns on the untreated PDMS regions. The fabricated prototype showed high-pressure sensitivity to the dynamic movement of an ant (0.025 g) placed on top. In addition, direct writing of LMs using a 3D positioning system, a dispensing syringe, and a needle could realize liquid patterns with a width down to 70 µm and a minimum separation of 200 µm on either flat or rough elastomeric substrates (Yoon et al., 2019).

#### 5.1.4 Capacitive-Based Microfluidic Tactile Sensors

Flexible capacitive sensors using embedded solid metal films (Lee et al., 2006) or carbon nanotubes (Engel et al., 2006) are known to be susceptible to failure in the form of fractures and fatigue. On the other hand, conductive liquids have been recommended as alternative components for forming flexible, durable capacitive sensing elements and wires. Liquid-based capacitive pressure sensors exhibited improved sensitivities over identically sized solid-based counterparts (Choi et al., 2015). As discussed earlier, the resulting increase in the sensor’s capacitance is proportional to the applied force that causes a reduction in the dielectric layer thickness.

Pressure mapping was addressed in microfluidic capacitive sensing. In one study, Wong et al. (2012) presented a flexible microfluidic capacitive force sensor with a 5 × 5 taxels array. Two PDMS active layers were orthogonally arranged: each one contained five parallel galinstan-filled microchannels. An air pocket PDMS layer was sandwiched between the two sensing layers to tune the sensor’s mechanical and electrical properties effectively. The proposed sensor could withstand forces up to 2.5 N under static mechanical loading tests and remained functional even after wrapping it around a small curvature surface. This sensor facilitated a decent spatial resolution of 0.5 mm, whereas the force sensitivity was very low, less than one pF N^−1^.

**FIGURE 6:**
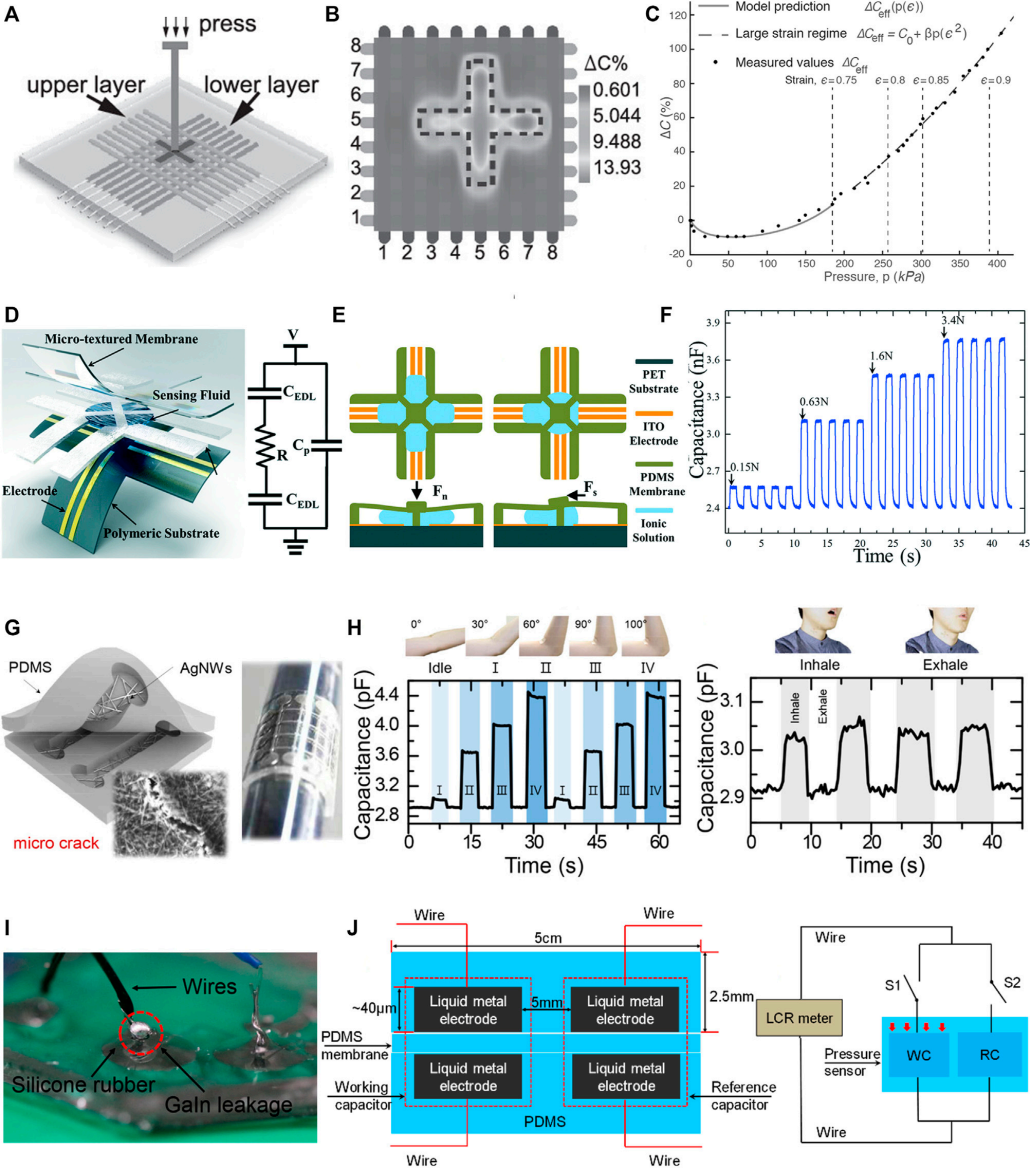
Microfluidic tactile sensing based on capacitance change. **(A)** Overview of an 8 × 8 sensing array based on liquid metals embedded in the elastomer. **(B)** Sensing pattern attributed to the capacitance change in the regions where external pressing was applied. **(C)** Comparison of the mutual capacitance between channels and laboratory measurements under force-controlled loading (Li et al., 2016c). Copyright 2016, AIP Publishing LLC. **(D)** Schematic illustration of the 3D microfluidic sensing structure and equivalent circuit diagram. **(E)** Schematic drawings of the operation principle under normal and shear force loads (in both top view and cross-sectional view). **(F)** Time-resolved sensor response to repetitive mechanical loads (Nie et al., 2014b). Reproduced with permission from the Royal Society of Chemistry. **(G)** Schematic showing lamination of two AgNWs-embedded PDMS panels and photographic image of a 4 × 4 stretchable sensor matrix attached to a steel bar of 1 cm diameter. **(H)** Variations in the capacitance of the crack-enhanced microfluidic sensor attached to arm joint and neck under bending and deep inhalation/exhalation motions, respectively (Ho et al., 2017). Reprinted with permission. Copyright (2017) American Chemical Society. **(I)** Leakage of the EGaIn liquid metal from the end of the microchannel under large pressure. **(J)** Structure of the double-capacitor sensor and equivalent circuit of the measurement (Zhang et al., 2019). Copyright (2019) MDPI.

With a similar design and goal, Li et al. (2016a) developed a microfluidic capacitive tactile sensor array based on multi-layer heterogeneous 3D structures of Ecoflex (Figure 6A). This sensor was redesigned to mitigate the effects of a non-monotonic regime present in an older design at low strains, where the value of capacitance first decreases and then increases after passing a threshold (Li et al., 2016b; Li et al., 2016c). Each cross‐point between the EGaIn-filled microchannels of the upper and lower layers acted as a separated capacitor, distinguishing the spatial distribution of applied forces with 2 mm resolution (Figure 6B). The passive and mechanically tunable intermediate layer enclosed air cavities and micropillar array geometric supports. After functional testing, the measured results showed excellent agreement with that from finite element analysis while conforming to flat or curved surfaces (Figure 6C).

Towards measuring shear forces, Nie et al. (2014b) proposed a novel tactile sensor design consisting of four PDMS sensing elements filled with an IL (Figure 6D). The sensor design incorporated a central force-magnifying bump that could deform the surrounding sensing elements either uniformly or differentially in response to normal or shear loads, respectively (Figure 6E). The ultrahigh-capacitive interface formed at the elastic ionic–electronic interface was utilized for flexible pressure-sensing, with substantially improved sensitivity of 29.8 nF N^−1^, tunable dynamic range up to 4.2 N, and relaxation response up to 12 ms. In Figure 6F, the reversibility of the sensor was reported under repetitive external loads. Besides, several types of actions were identified by a fingertip-mounted sensor.

Alternatively, Roberts et al. (2013) reported a microfluidic capacitive sensor that uses differential measurements in multiple EGaIn-based parallel-plate capacitor taxels for pressure and elastic shear sensing. The fabricated sensor was produced by a novel masked deposition process as an alternative to the injection-filling fabrication method. This fabrication approach allows for large-area planar geometries that are otherwise difficult to fill with needle injection. The sensor could detect two-directional shear displacements and normal pressures with resolutions of 500 μm and 5 kPa, respectively.

Fascinatingly, microfluidic capacitive tactile sensors can be integrated with other sensing principles for multi-modal tactile sensing devices. In one implementation, a single galinstan-based sensor, consisting of one upper channel and two lower channels symmetrically arranged in PDMS, achieved resistive-based pressure sensing and capacitive-based strain and curvature sensing (Zhou et al., 2020). In Noda et al. (2013)’s microfluidic sensor, the simultaneous sensing of tri-axial forces and stretches were enabled by capacitive and impedance sensing, respectively. Each force sensor unit consisted of 4 capacitors such that matching capacitance change profiles correspond to a normal force and non-matching profiles represent a shear force. While measuring the strain, the stretch-induced change in capacitance could be excluded from the force measurement. A follow-up work reported that force sensitivity could reach 0.0074 N^−1^ with an average stretch sensitivity of 3.2 strains (Noda et al., 2014). Furthermore, the capability of detecting the distribution of normal forces was demonstrated. This highly flexible and stretchable sensor shows promising potential for applications involving moving parts such as robotic joints.

Towards E-skin applications, Ho et al. (2017) demonstrated the fabrication of a transparent, stretchable capacitive pressure sensor based on fixing AgNWs on the microchannels’ bottom surface. Among five different sensing liquids, ethylene glycol (C_2_H_6_O_2_) was selected for its relatively low vapor pressure and good sensitivity. These microchannels were enhanced by microcracks and arranged in a crisscross shape (Figure 6G). An external loading, such as a pressure or a stretching strain, deforms the microfluidic layer between the two electrodes of the sensing cell, forcing the sensing liquid to penetrate the microcracks. Consequently, the interfacial liquid-electrode contact area increases, producing detectable capacitance variations in the sensor. The performance of the proposed microfluidic sensor was demonstrated by capturing substantial actions at the joint parts of the arms and legs and slight muscle movements on the neck and face (Figure 6H). Moreover, stress distributions during selectively pressing on a 4 × 4 sensor matrix were displayed as highlighted 2D color maps. Lastly, the proposed sensor stands promising for remote-sensing applications with the possibility of being integrated onto cylindrical tools such as endoscopes.

Under extreme loading, the working fluid is susceptible to leakage at the ends of microchannels (Figure 6I). In order to prevent that, Zhang et al. (2019) proposed injecting a leakage-free liquid electrode into the ends of the microchannels. The LM of Bismuth Indium Tin Alloy (Bi_32.5_In_51_Sn_16.5_), when added to a sandwich-structured sensor consisting of PDMS substrates, a pair of EGaIn electrodes, and silver-plated copper wires, could prevent leaking at extended measurement ranges with improved sensitivities. Additionally, the presented double-capacitor sensor can effectively reduce parasitic capacitance compared to the single-capacitor sensor (Figure 6J).

Microfluidic capacitive tactile sensing has successfully attempted wireless sensing applications. A flexible microfluidic device-based sensor built using galinstan has enabled wireless human motion monitoring (Munirathinam et al., 2020). Based on inductive coupling between the sensor antenna and an external readout coil, this sensor could provide real-time pressure measurements with up to a 10 mm working distance. The sensor was capable of identifying various bending angles of the wrist and index finger motion. With sulfuric acid treatment, the improved non-wetting characteristics of galinstan inside the microchannels achieve higher fluid velocity and faster sensor response.

#### 5.1.5 Other Microfluidic Tactile Sensors

There are several other emerging concepts for developing microfluidic-based tactile sensors. One example is the triboelectric-based microfluidic tactile sensors that are similar to the solid-state piezoelectric force sensors described earlier in this review. The triboelectric tactile sensing approach is based on the triboelectrification between two materials of different electron affiliations. When these two materials come into contact, the stronger electron affiliation material attracts electrons and thus becomes negatively charged, while the other material tends to lose electrons and becomes positively charged. When separated, the induced potential difference between positively and negatively charged substances will drive electrons to flow, firing an output signal (Lin et al., 2013). However, the triboelectric mechanism can only be used for dynamic pressure sensing as it requires nonstop movements for the output signal to be generated. While applying a static pressure, there will be no triboelectric output similar to the no-pressure state. Triboelectric-based pressure sensors have a simple structural configuration that is cost-effective even for large-area sensing and can be easily fabricated on various flexible substrates at low temperatures.

The liquid triboelectric pressure sensor presented by Shi et al. (2016b) shows great potential in biomedical applications. Based on the triboelectrification between DI water and PDMS, detection of dynamic pressure change was accomplished without any external power supply. A small PDMS disc was integrated on top for a better pressure transfer to the chamber. Accordingly, force sensitivity reached 0.0323 N^−1^. Capacitive sensing, as a complementary sensing mechanism of the prototype, allowed the static pressure changes to be measured. Several potential applications were demonstrated, i.e., monitoring microfluidic flow rate and human finger bending degree and frequency. Likewise, triboelectric nanogenerators based on LM electrodes embedded in an elastomer can function as wearable, elastic devices capable of transforming the mechanical deformation profile into electrical energy. Helseth (2018) featured interdigitated LM electrodes as triboelectric nanogenerators for mounting on human skin or other curved surfaces, wherein the elastomer can be simply conformed or stretched. Within the demand for second-life plastic waste in the current scenario of the circular economy, recycled plastics can be transformed into a microfluidic tactile sensor based on triboelectricity (Fang et al., 2018). Since being self-powered, the triboelectric sensing mechanism turns out to be particularly suitable for wireless sensing.

Another interesting example is the development of an optic-based microfluidic tactile sensor. Although most of the literature microfluidic examples have utilized electrical measurements to sense mechanical deformation, it is also possible to detect deformation through optical effects or light detectors. In an LM-based sensor utilizing diffraction of light, pressure-induced deformation of the elastomer microchannel forced the walls to buckle (Mohammed and Dickey, 2013). Correspondingly, a rigid oxide layer on the elastomer channel wall, formed after a plasma oxidation step to seal the microchannels, created a soft diffraction grating. The presence of the liquid metal conforming to the walls turns the diffraction effect on since light reflects back from the surface of the metal. An implantable microfluidic device optimized for self-monitoring of intraocular pressure of the human eye was described (Araci et al., 2014).

Moreover, an interesting microtubular-based microfluidic tactile sensor was developed, where the sensing approach can be simplified into soft tubular microfluidics, in which the sensing liquid is enclosed by a thin elastomeric tube (Xi et al., 2017a). A flexible capacitive strain sensor was formed by injecting liquid metal into two hollow elastomeric fibers intertwined into a helix (Cooper et al., 2017). This microfiber sensor provides a simple mechanism for creating large scalable torsion, strain, and touch sensors for pulse monitoring (Xi et al., 2017b) and smart textile applications (Yu et al., 2019).

Substantially, microfluidics is a type of liquid-state soft electronics that can simplify manufacturing, prototyping, assembling the device, and use. As evidenced by the earlier discussion, microfluidic sensors are more flexible than other conventional MEMS-based sensing devices, thanks to the liquid-based sensing elements and elastomeric structure of the sensors. Such unique sensing devices can be made in a variety of designs and offer a linear performance over a wide temperature range. Furthermore, the sensitivity and working range can be controlled by intentionally changing the dimensions of the microchannels and the structural design and the base material of the sensor. In addition, microfluidic tactile sensors demonstrate a remarkable ability to deform and adapt to the shape of the surface of installation. They also show high sensitivity and fast response time comparable to that of the conventional tactile sensor. Moreover, the transparent nature of commonly used elastomeric materials as the base for this type of sensor gives it an extra advantage over its conventional counterparts. With all these pluses, microfluidic-based tactile sensors are more favorable for many types of biomedical applications. Table 4 comprehensively summarizes recent innovative microfluidic tactile sensors in terms of the working principle, liquid, substrate, measurement type, sensitivity, and demonstration.

**TABLE 4:** Comprehensive summary of the microfluidic-based tactile sensors (arranged chronologically).

Reference	Principle	Liquid	Substrate	Measurement type	Sensitivity	Demonstration	Remarks
Tseng et al. (2009)	Impedance	Sodium chloride (NaCl)	PDMS on Polyimide (PI) substrate	Normal force	6.06 mV N^−1^	—	Mimicking slow-adapting receptors of human skin
Gutierrez and Meng (2010)	Impedance	DI water	Parylene C	Normal force	—	Mechanotransduction along interconnected channels	Capable of providing actuation through electrolysis of water
Park et al. (2010)	Resistive	Eutectic Gallium–Indium (EGaIn)	Ecoflex	Normal force and strain	—	Artificial skin for pressure and strain sensing up to 25 kPa and 250%, respectively (Park et al., 2012)	Control over working range and type of measurement (pressure vs. strain)
Noda et al. (2010)	Resistive	1-ethyl-3-methylimidazolium ethyl sulfate	PDMS	Normal force and strain	—	Detection of contact forces on curved surfaces	Strain compensation for an independent contact force measurement
Kramer et al. (2011b)	Resistive	EGaIn	PDMS	Normal force	—	Typing ‘HELLO WORLD’ using the keypad	Accommodating twelve keys in ≈700 μm of the total thickness
Shi et al. (2012)	Resistive	Coollaboratory Liquid Pro	PDMS	Normal and shear forces	—	—	Followed by hysteresis analysis (Shi et al., 2016a)
Wong et al. (2012)	Capacitive	Galinstan	PDMS	Normal force	—	—	Using a liquid metal-based internal circuitry
Vogt et al. (2012)	Resistive	EGaIn	Ecoflex	Normal and shear forces	Up to 37.0 mV N^−1^	—	Utilizing a force-post to capture shear forces
Hammond et al. (2012)	Resistive	EGaIn	PDMS	Normal force	—	—	Localizing contact with a sub-millimeter resolution
Roberts et al. (2013)	Capacitive	EGaIn	Ecoflex	Normal and shear forces	—	Smart glove for measuring the friction and pressure of the palm area	Fabricated by a novel masked deposition process
Arabagi et al. (2013)	Resistive	EGaIn	PDMS	Normal force	—	Miniaturized soft robotic tip sensor	Providing information about the angle of contact
Cheng et al. (2013)	Resistive	1-Ethyl-3-methylimidazolium dicyanamide	PDMS on Pyrex	Normal force	—	Measuring the spatially varying elasticity of a heterogeneous material	Detecting distributed static and dynamic loads
Noda et al. (2013)	Capacitive	1-Butyl-1-methylpyrrolidinium tetracyanoborate	SH-9555 silicone rubber	Normal and shear forces and strain	Up to 0.0074 N^−1^	—	Measuring tri-axial forces on movable components, i.e., joints (Noda et al., 2014)
Yang et al. (2014)	Resistive	1-Ethyl-3-methylimidazolium dicyanamide	PDMS on Pyrex	Normal and shear forces	—	—	Followed by a demonstration of torque measurement about the *Z*-axis (Yang and Hao, 2015)
Nie et al. (2014b)	Capacitive	1-Ethyl-3-methylimidazolium tricyanomethanide	PDMS on PET substrate	Normal and shear forces	29.8 nF N^−1^	Fingertip sensing	Tunable dynamic range and relaxation time up to 4.2 N and 12 ms, respectively
Codd et al. (2014)	Resistive	EGaIn	PDMS	Normal force	—	Integrated on an endoscope operating sheath	Followed by sensitivity and biocompatibility enhancements (Arabagi et al., 2016)
Yang et al. (2015)	Resistive	1-Ethyl-3-methylimidazolium dicyanamide	PDMS on Pyrex	Normal force	—	Tissue phantoms palpation for identifying abnormalities	Palpating mice tumor tissues (Yang et al., 2016b)
Chossat et al. (2015)	Impedance	1-Ethyl-3-methylimidazolium ethyl sulfate	Ecoflex	Normal force	—	Detection of magnitudes and locations of surface contacts	Injecting the IL through a silicon mesh layer for a complete filling of microchannels
Kenry et al. (2016)	Resistive	Graphene Oxide nanosuspension	Ecoflex on PDMS substrate	Normal force	0.0338 kPa^−1^	Detection of hand gestures and muscle-induced motions	Performing flow leakage tests on different flexible material assemblies
Shi et al. (2016b)	Triboelectric	DI water	PDMS on PET substrate	Normal force	0.0323 N^−1^	Flow rate and finger motion monitoring	Detecting the dynamic pressure change without external power supply
Li et al. (2016a)	Capacitive	EGaIn	Ecoflex	Normal force and strain	—	Mounting on a robotic or human finger	Remarkable dynamic response and conformability on curved surfaces
Yeo et al. (2016a)	Resistive	EGaIn	Ecoflex on PET film	Normal force	0.002−0.02 kPa^−1^	Foot stomping, chair rolling, and car wheel rolling over the sensor	Sensitivity enhancement with parallel arc structure (0.05 kPa^−1^) (Yeo et al., 2016b)
Yeo et al. (2017)	Resistive	EGaIn	Ecoflex on PET film	Normal and shear forces	0.06 kPa^−1^	Braille reading	Sensing surface features of round/sharp edges and hard/soft materials
Gao et al. (2017)	Resistive	Galinstan	PDMS	Normal force	0.0835 kPa^−1^	Heart-rate monitoring and sensorized PDMS glove for tactile feedback	Applying a Wheatstone bridge circuit for temperature self-compensation
Ho et al. (2017)	Capacitive	Ethylene glycol and four other sensing liquids	PDMS	Normal force and strain	Up to 0.021 kPa^−1^	Substantial and slight muscle movement sensing	Microcrack-enhanced PDMS microchannels arranged in a crisscross fashion
Yin et al. (2017)	Resistive	EGaIn	PDMS	Shear force	0.088 N^−1^	Sensing dynamic shear force, vibration, and slippage (Yin et al., 2018)	The design provides space for integration with a normal force sensor
Yoon and Chang (2017)	Capacitive	1-Butyl-3-methylimidazolium tetrafluoroborate	PDMS	Normal force	1.01 × 10^−3^ kPa^−1^ at 6 wt% CNTs	Sensing habitual hand motions and temperature/pressure applied to a bottle	Temperature sensitivity up to 3.46% °C ^−1^ at 6 wt% CNTs
Liu et al. (2018)	Resistive	EGaIn	Polyacrylamide (PAA)‐alginate hydrogel	Normal force and strain	100 Pa	Measuring fingertip presses	Reusing dehydrated and dysfunctional hydrogel electronics by hydration
Kim et al. (2019)	Resistive	Galinstan	Dragon Skin 10	Normal force	0.158 kPa^−1^	Wireless monitoring of epidermal pulse and heel pressure	Monolithically integrating 3D‐printed PLA microbumps with the microchannel
Zhang et al. (2019)	Capacitive	EGaIn	PDMS	Normal force and strain	Up to 0.45 MPa^−1^	—	Filling the ends of the microchannels with BiInSn for leakage prevention of GaIn under large pressure
Wang et al. (2020)	Resistive	Galinstan	PDMS	Normal force	0.08 kPa^−1^	Fingertip grasping force and temperature sensing	Temperature sensing sensitivity of 0.41% °C ^−1^ between 20 and 50 °C
Zhou et al. (2020)	Resistive	Galinstan	PDMS	Normal force, strain, and curvature	—	—	Installing an additional metal shell to increase the pressure-sensing range
Low et al. (2020)	Resistive	EGaIn	Ecoflex	Normal force	—	Gait monitoring	Wireless data transmission to a smartphone
Wu et al. (2020)	Resistive	Galinstan	PDMS	Normal force	—	Sensing the dynamic movement of a small sphere and an ant	Introducing laser-induced selective adhesion transfer for liquid metal patterns
Munirathinam et al. (2020)	Capacitive	Galinstan	PDMS	Normal force and strain	5 kHz/mmHg	Wrist and index finger motion monitoring	Appling an inductor-capacitor (LC) resonant circuit for wireless readout method

While the microfluidic-based tactile sensing approach is still in the developmental phase, its potential worthiness in MIS tactile sensing was demonstrated in a few studies discussed earlier. Due to low cost and ease of fabrication, microfluidic sensing devices can be disposable and easily integrated with the MIS tools in a plug-and-play format. Nowadays, the field of microfluidics is emerging with various applications in biomedical sciences; therefore, one can take advantage of advances in microfluidics to invent new principles and soft devices for the MIS field. Besides, microfluidic-based sensors can be further miniaturized in size with the advancements in microfluidics fabrication techniques, hence becoming more suitable for integrating the tip of the laparoscopic or other MIS tools without compromising the function of its surface. Furthermore, multi-layered 3D sensing devices based on microfluidics can be envisioned for multiplexed tactile sensation. Eventually, the microfluidic sensing approach is yet to be further emerged in the near future to realize unmet goals in the MIS tactile sensing field.

The remaining challenges of microfluidic sensors in MIS include fluid leakage at the injection ports, failure of the elastomeric structure of the sensor, the mechanical mismatch between the liquid and the polymer, the long-term stability of microfluidic sensors, wire bonding, and compatibility with the other electronic components on the surgical tool. As for any clinical and medical tool, the biocompatibility of the microfluidic sensors is a primary concern when considering MIS applications. Therefore, more attention to the durability of the sensor’s packaging and injection ports sealing is required to prevent any leakage of working liquids. More durable sensors are needed for using sensing liquids of higher toxicity levels. The ultimate goal is to employ proper working liquids made of nontoxic, biodegradable, and biocompatible compounds, such as biocompatible ionic liquids (Gomes et al., 2019). Additionally, working liquids should cause no allergic reactions, be harmless to internal body organs and tissues, and have a proper pH level. While dealing with LMs, the oxidation of LMs and their tendency to corrode other metals should be prevented to avoid any increase in the overall resistance or performance degradation (Zhu et al., 2020).

### 5.2 Imaging-Based Tactile Sensing

Recent rapid advances in computer vision and machine learning have drawn increasing attention towards imaging-based tactile sensing, also known as vision-based sensing concepts. This subclass of optical-based sensors is also boosted by the accessibility to low-cost image sensors with superior performance and miniaturized size. Generally, imaging tactile sensors are composed of three major elements: a tactile membrane/skin, an imaging device, and a computer (Shimonomura, 2019). The tactile membrane, upon which external mechanical loadings are exerted, functions as a physical contact-light conversion medium. The induced visual change of that skin serves as a sensing transducer. Meanwhile, the design and stiffness/rigidity of the membrane vary depending on the adopted sensing method and the desired working range. In the case of being elastomeric, the tactile skin would undergo deformation in compliance with the applied force. Underneath the skin lies a device, referred to as a camera, consisting of an image sensor and optical system. A tactile sensor employing a three-color camera can obtain detailed tactile information with a high spatial resolution (Kamiyama et al., 2004). Other advanced imaging system technologies, i.e., depth cameras (Alspach et al., 2019) and dynamic vision sensors (Baghaei Naeini et al., 2020), have been employed for the same goal of tracking externally induced visual features and providing high-resolution information about the deformation of a soft elastomeric surface. In addition, event-based cameras can measure contrast change in time and give a reading of intensity temporal change at each pixel (Kumagai and Shimonomura, 2019). Tactile imaging sensors might also be integrated with an illumination system, e.g., light-emitting diodes, to provide higher clarity of the visual changes. After capturing live imaging of the membrane, it is possible to derive a prediction of the properties of the force applied to the sensor’s surface by training a learning algorithm with an extensive amount of data.

A typical drawback of the camera-based sensors is the bulkiness of their main sensing unit since the sensing skin must be stacked above the camera (Kamiyama et al., 2004). Moreover, additional space is required between the camera lens and the soft tactile surface due to the minimum focal distance of commercial cameras. Even with close-focus lenses, placing the soft surface close to the camera reduces the field of view. Meanwhile, such sensing principle poses significant advantages of, but not limited to, high spatial resolution, measurement area control through an optical system, isolation of the camera, and usage of computer algorithms (Shimonomura, 2019). Imaging-based tactile sensing allows force measurement over large areas with high spatial resolution while minimizing the amount of wiring required by the conventional electrical sensors. With the advances of imaging sensors, a larger number of sensing points and finer pixel pitches can be easily realized (Duke et al., 2019). The exposed size of the skin’s surface can be controlled by imaging lenses, hence indicating the resolution of the tactile sensor.

In one study, a tactile imaging sensor with translucent hollow cylinder elastomer with eight miniature conical legs was developed with normal and shear force measuring capabilities (Li et al., 2018). The contact pattern and force magnitude were measured *via* the transparent elastomer and the change of eight feet, respectively. Normal force acting in the sensor induces identical visual changes at the eight conical feet, whereas lateral moment induces uneven changes relevant to the direction of shear force.

Several approaches have been established for imaging-based tactile sensing. In one approach, trackable patterns, such as markers or particles, were embedded in the elastic surface. The induced movement of these markers is directly related to the strain field of the material and can therefore be used to reconstruct the external force distribution on the surface. With respect to the motion of markers, circular shapes are nondirectional and provide a uniform number of events, such as a change in brightness. In contrast, the directional movements of polygonal markers such as triangles and rectangles provide a different number of events. With a higher density of markers, better spatial uniformity of response can be achieved (Yamaguchi and Atkeson, 2019). Furthermore, the size of markers affects the accuracy of tracking.

The soft elastomer layer of Kumagai and Shimonomura’s (2019) tactile sensor had a hemispherical shape of 40 mm diameter and carried 361 white markers on its backside. The sensor exhibited 0.5 ms temporal resolution over 128 × 128 sensing pixels. While the sensor stands promising for detecting information about the object in contact, i.e., slip and position, it is difficult to get the absolute and accurate orientation of the object from the sensor reading after post-processing the captured movement of markers.

**FIGURE 7:**
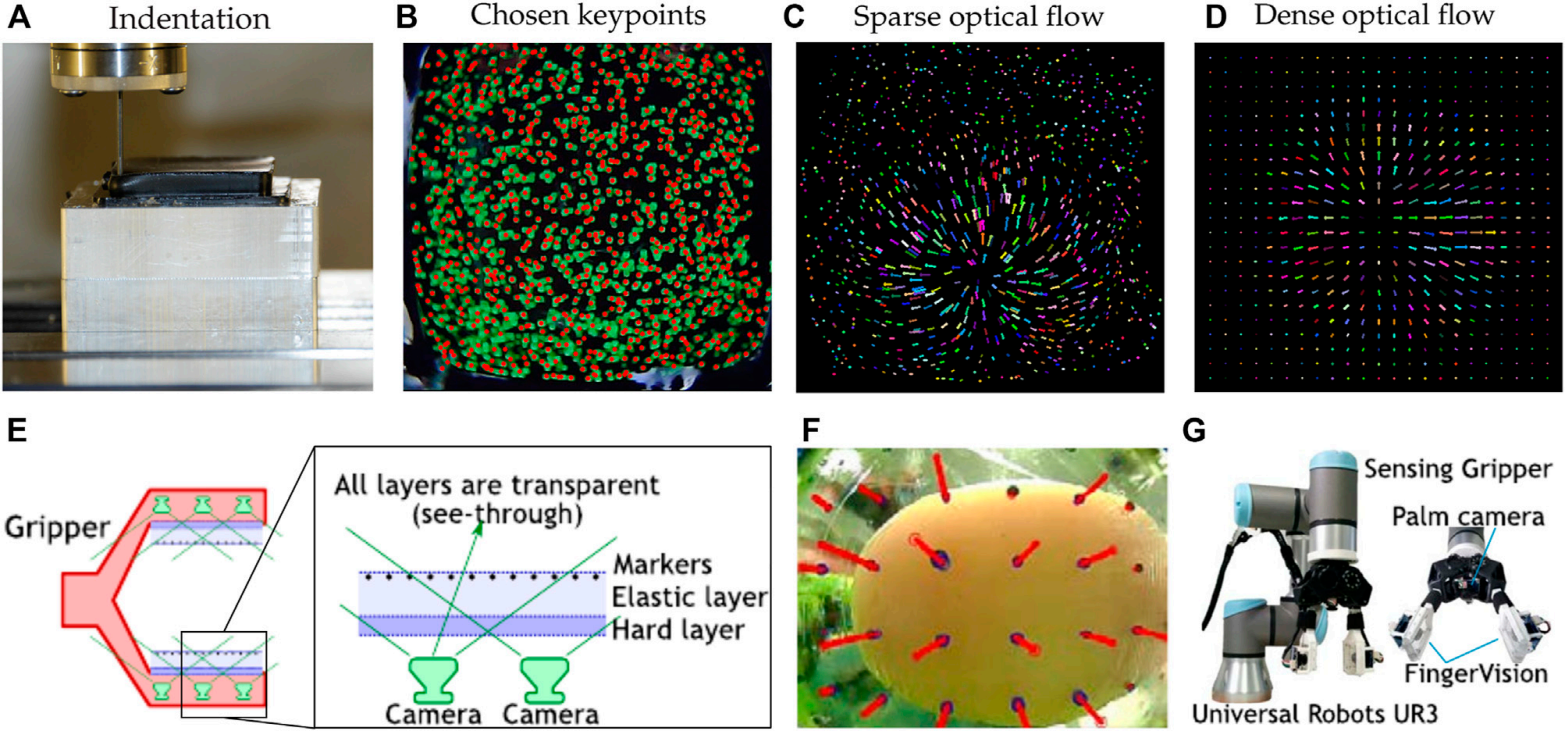
Imaging-based tactile sensing. **(A–D)** Example of a tactile image sensor using a camera based on the marker displacement method. **(A)** The data collection setup, showing the prototype under the automated milling machine. **(B)** The original image segmented into the different markers in red, using the watershed algorithm. Two generated optical flows: **(C)** the key points tracked through Lucas Kanade optical flow and **(D)** the Dense Inverse Search (DIS) algorithm computes the dense optical flow at each pixel on the resulting image (Sferrazza and D'Andrea, 2019). Copyright (2019) MDPI. **(E)** Installation sketch on a robotic gripper and conceptual design of FingerVision system. **(F)** Example of marker movements when a normal force is applied. **(G)** UR3 robot with FingerVision (Yamaguchi and Atkeson, 2019). Copyright (2019) World Scientific.

Sferrazza and D’Andrea (2019) studied the influence of markers’ placement at different depths inside the soft sensing surface. The results showed increased robustness to noise with markers sitting closer to the camera, i.e., deeper in the soft material. However, a counteracting effect of deeper markers was the inducement of smaller displacements compared to markers close to the surface of contact. The homogeneous spread of markers within the soft sensing surface yielded an advantageous trade-off between robustness to noise and sensor threshold. Figure 7A shows the developed prototype having a sensing surface of 30 × 30 mm and a thickness of 37 mm. Two learning architecture algorithms, namely, watershed and dense inverse search, were applied to construct optical flow images and estimate the normal force distribution (Figures 7B–D). Force resolution of 0.06 N in the 480 × 480 pixels sensor was achieved. However, this work was limited to normal force measurement with a maximum of 1 N.

In an extension study, the field of view was increased by placing four cameras next to each other (Trueeb et al., 2020). The cameras were equipped with close-focus lenses, which helped in reducing the overall thickness down to 17.45 mm. With the aid of an end-to-end deep neural network, this work illustrated the possibility of training the most time and data-consuming part of the network on a subset of the surface, e.g., 3 out of 4 cameras. Consequently, data collecting and training times can be reduced effectively. Additionally, multiple cameras were employed to reconstruct the 3D displacement of markers in a soft tactile muscularis (Duong et al., 2019). Bio-inspired sensors, having skins mimicking the structural details of human fingertips, demonstrated the concept of object edge encoding (Chorley et al., 2009).

Another approach for imaging tactile sensing employs a light conductive plate. Here, the tactile skin can be either rigid or soft and mostly made out of transparent acrylic, glass, or silicone rubber. The translucent light conductive plate is illuminated with a light that satisfies the internal reflection condition, where the light incident angle θ is larger than the critical angle $\theta_{c}$ (Shimonomura et al., 2016). This condition is observed when light from a medium with a larger refractive index $n_{1}$ enters another medium with a smaller refractive index $n_{2}$ . The critical angle can be represented as follows:

$$\theta_{c}=\sin^{-1}\frac{n_{2}}{n_{1}}.$$

When the critical angle increases by the contact with an object of refractive index $n_{3}\;$ larger than $n_{2}$ , the incident angle can no longer fulfill the total internal reflection condition. Correspondingly, the trapped light inside the conductive plate goes out of the plate at the contact point and reflects the object. Such scattering of light is captured by the camera. Infrared illumination light can separate the incident light to the camera into the visible light from outside the conductive plate and the infrared light occurring by contact. Ultimately, a light conductive plate of higher refractive index material is desirable for higher sensing sensitivity.

Shimonomura et al. (2016) demonstrate a combined tactile and proximity sensing using a light conductive plate and three cameras. One camera equipped with a visible light cut filter was used to capture the light scattered by the object contact, whereas the two other cameras equipped with infrared cut filters and oriented to slightly different viewpoints (compound-eye) were used for calculating the distance to the object. Robotic motions of searching, approaching, and grasping were fully controlled based on information obtained from the proposed device only. Since the pixel value in the contact image depends on the optical characteristics of the object’s surface, it is challenging to measure contact force without identifying the object first. To measure applied force, a bumpy elastomer cover can be placed on top of the light conductive plate so that parameters of the refractive index and spectral reflectance are specified (Kamiyama et al., 2004). When a stronger force is pressed against the surface, the microscopic increase in contact area amplifies the captured brightness. Yet, the measurement is limited by the number of bumps.

Another approach to generating trackable feature changes is by employing a reflective membrane at the back of the elastomeric tactile surface. Objects making contact with the sensor surface induce deformations. Correspondingly, a shading image reflecting these deformations appears in the captured image from the camera. In one demonstration, Johnson and Adelson (2009) placed a clear elastomer covered with a reflective skin on top of a camera to construct a tactile imaging sensor. When an object was pressed on the sensor, the distorted skin duplicated the object’s surface shape and texture and appeared as a replica when viewed from behind. Imaging with illumination from red, green, and blue light sources at three different positions was fed into a photometric stereo algorithm to reconstruct the surface.

Saga et al. (2007) developed a tactile sensor utilizing transparent silicone rubber skin as a flexible mirror surface combined with the optical lever technique. This technique magnifies the observed displacement through the characteristics of reflection. Underneath the skin, an inversed prism structure was accommodated, having a patterned surface and capturing surface. The system detected the deformation of the tactile surface by measuring the displacement *via* a reflection image from the mirror surface. Eventually, the design of a thinner device using a saw-shaped rubber was presented. The loss of information at the point of contact with a broad planar shape remains a drawback of the proposed design. Although detecting the shape and texture of objects was accomplished, this imaging tactile sensing approach remains incapable of providing force measurement.

Recently, dual-modal imaging-based tactile sensors have been introduced to robotic applications. Fang et al. (2018) proposed a tactile sensing device that combines the two imaging approaches of reflective membrane and marker displacement. During robotic hand grasping, the sensor was capable of measuring the distribution of applied force vectors and recognizing the shape and texture of the object in contact (Fang et al., 2018). The developed tactile sensor could be easily mounted on robotic hand fingertips. Similarly, Nozu and Shimonomura (2018) proposed a tactile imaging sensor that provides in-hand object localization and force measurements. The usefulness of the sensor was demonstrated by performing two robotic arm tasks of bolt insertion and tightening. Such tactile sensing devices, with force and surface texture sensing ability, possess a promising potential for other robotic applications, including RMIS.

In addition to tactile sensation, the vision of a nearby object can be achieved by utilizing transparent sensor skins. Yamaguchi and Atkeson (2019) developed a multimodal tactile sensor called FingerVision (Figure 7E). As the embedded camera sees through the translucent skin, the system was able to obtain information about the object’s distance, location, pose, size, shape, and texture. Besides, the sensor can sense distributions of force and slip based on the markers method (Figure 7F). The ability to estimate torque information was demonstrated by combining multiple marker measurements. The trade-off between the resolution and the surface transparency associated with the density of markers was highlighted. Lastly, tactile behaviors were explored *via* universal robots named Baxter and UR3 (Figure7G). Depth-sensing cameras allow for touch detection on non-flat and non-instrumented interactive surfaces. Moreover, information about the shape of the users’ hands and arms above the surface can be exploited in a useful manner, such as determining hover states and whether multiple touches are from the same arm (Wilson, 2010).

While tactile sensors are recommended to be durable against frequent contact with objects in practice, tactile imaging-based sensing structure requires that the actual measurement device, i.e., the camera, be separated from the contact point. As an advantage, it is relatively easy to replace or repair the elastic portion of the sensor in case it breaks. In the aim towards developing MIS tactile sensors that fully imitate human fingertips, imaging tactile sensors employing high-resolution cameras come to realize tactile sensing ability far beyond that of humans. An extra advantage is that while receiving force feedback, surgeons can have a live view of the grasped region of the organ if the sensing skin is transparent. Imaging-based tactile sensors can only get smaller and smaller with the developments of cameras that are as small as a grain of sand, e.g., OV6948 CMOS chip (OmniVision Technologies, Santa Clara, CA). Eventually, MIS tools incorporated with cameras can go smaller and thinner, effectively entering blood vessels (Nicholas et al., 2021). Furthermore, imaging-based sensors are very promising for robotic manipulation tasks requiring rapid, responsive grasping and preventing slippage, as multi-axial force measurement can be realized with multiplexing of various static and dynamic loadings. Hence, RMIS can be much enhanced by incorporating imaging-based tactile sensors on the surgical end effectors of the robot.

The progress in microfluidic- and imaging-based tactile sensing developments opens new directions for MIS-related tactile research. The combination of imaging and optical sensing principles using cameras for collecting images of the tactile surface and capturing the change in the light intensity of optical fiber seems to be an interesting topic worthy of investigation. Moreover, incorporating the transparent skin layer of the tactile imaging sensor with microchannels stands promising towards achieving multimodal tactile sensing. Many other hybrid sensors can be inspired for multimodal tactile sensing applications. Table 5 highlights the major advantages of both emerging tactile sensing technologies, such as better flexibility and transparency, compared to conventional MEMS-based tactile sensors for MIS applications.

**TABLE 5:** Comparison between the conventional and emerging tactile sensing technologies for MIS.

Classification		Flexibility	Fabrication	Biocompatibility	MIS adaptability	Transparency
Conventional	MEMS-based	Low	- MEMS technology	Mostly biocompatible	Easy	Low
			- Photolithography			
Emerging	Microfluidic-based	Very high	- 3D printing	Biocompatible elastomers and microchannel’s sealing	Easy	High
			- Molding			
			- Liquid injection			
	Imaging-based	Low	Image sensor:	Biocompatible sensing skin	Moderate	High (Partial)
			- CMOS technology			
			Sensing skin:			
			- 3D printing			
			- Molding			

## 6 Discussion

The recent advances in sensing technologies and robotics have fueled the development of tactile sensors, especially for MIS. With the aid of tactile sensors, MIS tools can become more valuable in surgical practices and achieve better medical outcomes. Besides, laparoscopic graspers and probes equipped with tactile sensors are favorable for the training of novice surgeons (Özin et al., 2019). In this review, we discussed the literature on principles and applications of tactile technologies in MIS tactile sensing. We also highlighted the achievements of conventional sensing technologies and the potential of emerging technologies to take the lead towards low-cost, high-performance tactile sensors. Our motivation was to find the main reasons behind the delay of sensorized MIS instruments in making their way to the commercial level.

Throughout our study, we investigated several technologies and principles of tactile sensing. Among others, electrical-based tactile sensors have dominated the field of MIS and MIRS tactile sensing. Piezoresistive materials, in specific, are the most widespread in electrical-based sensing. This is due to the nature of piezoresistive materials, which facilitate the microfabrication of flexible and compliant thin films. Additionally, piezoelectric sensors are commonly used for MIS tactile sensing applications. No additional power supply is required since piezoelectric sensors generate their own voltage. Capacitive sensing is the core of several tactile sensors employed for laparoscopic graspers and probes. They show better stability and increased sensitivity compared to their electrical counterparts. In contrast to electrical sensors, optical-based sensors make an estimation of the applied force from the mechanically induced changes in properties of the light passing through optical fibers; hence, they are electrically passive. Each of these conventional tactile sensing techniques possesses its own advantages and suffers from certain limitations. Creatively, different technologies can be combined into a multimodal sensing system aiming towards measuring the various tissue-tool contact parameters with increased efficiency and reliability.

From the literature analysis presented before, it is indisputable that conventional tactile sensing technologies have realized several prototypes capable of measuring the different types of forces involved during MIS practices. Nevertheless, most of these studies remained at the research level and did not make it to actual practices, as evidenced by the available laparoscopic instruments in the market. Therefore, we pointed up the promising potential of emerging tactile sensing technologies. The recent developments in microfluidic-based tactile sensors promise to improve the feasibility and flexibility of force and tactile sensing. These microfluidic soft sensors can improve the tissue grasping and manipulation tasks during MIS and MIRS by conforming to the surface of the jaw to increase contact friction, allowing stable grasps with smaller exerted forces, and enabling palpation to determine the geometry, mechanical properties, and position of the tissue in contact with a surgical instrument. While using biocompatible elastomeric structure, a proper sealing of the working liquid is a major concern for MIS applications since its leakage might cause health problems. Future work should focus on developing biocompatible working liquids for MIS-oriented microfluidic tactile sensors. Furthermore, solid electrical wires can be replaced by containing LMs in long, thin polymer tubes. Flexible and stretchable wires have been achieved accordingly (Zhu et al., 2013; Matsubara and Ota, 2019). Besides, microfluidic tactile sensors have a golden opportunity to be made compatible with MRI, as seen in other existing MRI accessories utilizing LMs (Port et al., 2020).

Imaging-based tactile sensing, although being a bit bulkier in size, exhibits higher resolution and faster response time since the facility of adopting cameras with next-generation specifications. While the typical drawback of the field of view limits the miniaturization of such tactile sensing technology, it can be overcome by developing larger image sensors that can span areas up to the size of the sensing elastomer. Besides, the sensing skin itself can be used as optics for light focusing and beyond. Additionally, the sensing elastomeric surface of the imaging tactile sensors can accommodate microchannels for microfluidics/imaging-based multimodal tactile sensors. Flexible image sensors are another way to achieve flexible imaging-based tactile sensors (Simone et al., 2020). Future investigations concerning these emerging tactile sensing technologies are expected to revolutionize the development of low-cost, high-performance MIS force sensors.
